# The Perizonium Ultrastructure, Divided Apical Pore Fields, Various Pore Occlusions and Visible Intermissio of *Cymbella* (Bacillariophyceae) with Descriptions of Four New Species

**DOI:** 10.3390/plants13131851

**Published:** 2024-07-05

**Authors:** Bin Yang, Bing Liu, Saúl Blanco, Patrick Rioual

**Affiliations:** 1 College of Biology and Environmental Sciences, Jishou University, Jishou 416000, China; yangbin02032024@163.com; 2 Laboratorio de Diatomología, La Serna 58, 24007 León, Spain; sblal@unileon.es; 3 Key Laboratory of Cenozoic Geology and Environment, Institute of Geology and Geophysics, Chinese Academy of Sciences, Beijing 100029, China; prioual@mail.iggcas.ac.cn; 4 CAS Center for Excellence in Life and Paleoenvironment, Beijing 100044, China

**Keywords:** apical pore fields, cymbelloid, initial valve, perizonium, synapomorphy

## Abstract

The initial valves of two *Cymbella* species are observed under a scanning electron microscope, and the perizonium ultrastructure of *Cymbella* is revealed for the first time. The perizonium is composed of alternate nodes and internodes and lacks transverse perizonium bands. Four new species, *Cymbella apiculatophora* sp. nov., *C. hunanensis* sp. nov., *C. juglandis* sp. nov. and *C. menyuanensis* sp. nov., are described using light and scanning electron microscopy based on epilithon samples collected from rivers in Hunan and Qinghai Provinces, China. *Cymbella menyuanensis* is a typical *Cymbella* species that closely resembles species in the group around *C. cymbiformis* Agardh, the type species of the genus. *Cymbella apiculatophora* is similar to *C. sinensis* Metzeltin & Krammer, while *Cymbella hunanensis* is closer to the *C. hustedtii* Krasske group. The last species, *C. juglandis*, has a cymbelloid valve outline, an obscured intermissio, internal occlusions of the areolae, dorsally deflected distal raphe fissures and a divided apical pore field at each apex, and it does not appear to belong to any group. In addition, new observations on *C.* cf. *excisiformis* Krammer and *C. hustedtii* are reported. The current concept of the genus *Cymbella* does not represent a monophyletic group as shown by molecular phylogenetic analyses. However, these analyses are still at the preliminary stage and are not yet sufficient to support a complete revision of the genus. Thus, although extremely diverse ultrastructural features are observed in the six *Cymbella* species investigated in this paper, we prefer to keep them within *Cymbella* at this moment for the sake of nomenclatural stability.

## 1. Introduction

Species of the genus *Cymbella* C. Agardh (type species: *C. cymbiformis* C. Agardh) are biraphid diatoms, characterized by dorsiventral valves, dorsally deflected distal raphe fissures, a hidden intermissio, the presence of apical pore fields (APFs) and stigmata [1]. In addition to the above characteristics, the type species, *C. cymbiformis*, also possesses areolae with internal occlusions [1]. According to the AlgaeBase website [2], the genus *Cymbella* currently includes 372 valid species names, 173 accepted varieties and 56 accepted formae. From these *Cymbella* taxa, we can see extreme diversity of morphological structures. Here, we focus on three characters in *Cymbella*: the APFs, pore occlusions and intermissio.

Liu et al. [3] defined two types of APFs in the genus *Cymbella*: type I is a complete APF located in an uninterrupted area; type II corresponds to an APF divided by the distal raphe fissure into two unequal areas, a larger ventral one and a smaller dorsal one. These authors only listed a few species with the type II APFs. Here, based on a more systematic review of illustrations published in the literature, we propose a list 16 *Cymbella* taxa possessing type II APFs (Table 1). For example, Lee et al. [4] thoroughly examined *C. orientalis* J.H. Lee and clearly showed that this species has type II APFs. Similarly, Krammer [1] provided an SEM image showing that *C. hustedtii* Krasske also has type II APFs. Unfortunately, neither Lee [4] nor Krammer [1] discussed the structure of the APFs. The first mention of type II APFs was most likely made by Rodionova et al. [5], who stated that “[APFs] are divided by dorsally deflected terminal endings in *C. hustedtii* and *C. orentalis*…”.

Regarding the occlusions of the internal apertures of APFs, Krammer [13] termed these occlusions “costal constructions”. For *Cymbella fontinalis* Bahls, Bahls [14] stated that “internally, each column of poroids in the apical pore field is partially covered by a knobby jointed ridge of silica” and thus provided the first detailed description of the internal occlusions of the APFs. Later, Liu et al. [15] described these occlusions as “an undulate flap-like silica strip above internal apertures of each row of foramina, but not occluding internal apertures completely”. The same group of authors also found similar occlusions in three *Delicatophycus* species [3].

We checked the references on *Cymbella* published since 1970 and found at least eight taxa for which the internal occlusions of the areolae are clearly illustrated (Table 2). These occlusions are simple: solid closing plates, either rounded or reniform, developed from a strut attached to the lumen wall of the areola.

The intermissio (i.e., the gap between the two internal proximal raphe endings) is hidden by a silica hood in most *Cymbella* species. However, some *Cymbella* taxa have a clearly visible intermissio. We have found at least four species with this character: *C. bourrellyi* Maillard ex Moser, Steindorf & Lange-Bertalot [18], *C. cognata* [5], *C. hustedtii* [1] and *C. subleptoceros* Krammer [1].

To our knowledge, the ultrastructure of the perizonium in *Cymbella* has never been illustrated in the literature. In this paper, we first describe four new species of *Cymbella* following the criteria and the characteristics of the type species of *C. cymbiformis* as provided by Krammer [1]—dorsally deflected distal raphe fissures, presence of apical pore fields and internal occlusions of the areolae—and provide new observations on *C.* cf. *excisiformis* Krammer and *C. hustedtii*. Then we provide preliminary observations on the perizonium of two *Cymbella* species. Finally, we analyze, summarize and discuss the results.

## 2. Results and Discussion

### 2.1. Results

**Figure 1:**
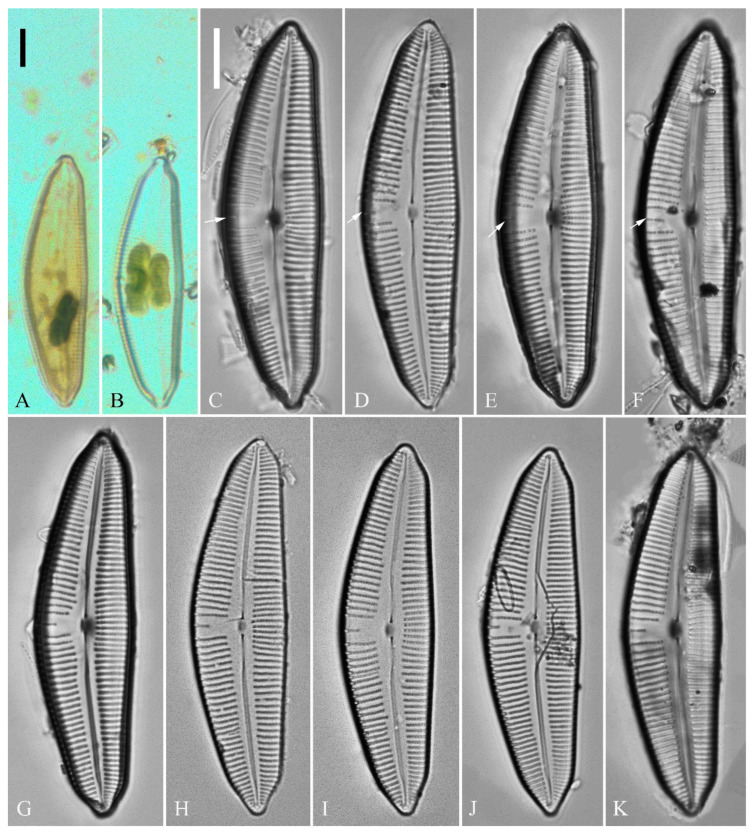
*Cymbella apiculatophora* sp. nov., LM. (**A**,**B**). Two uncleaned cells. (**C**–**K**). Nine valves showing a size diminution series; note one shortened stria on the dorsal middle part (arrows in **C**,**D**,**E**,**F**, respectively) and the apiculate apices. (**I**). Illustration of the holotype specimen. Scale bars (**A**,**B**) = 10 μm, (**C**–**K**) = 10 μm.

**Figure 2:**
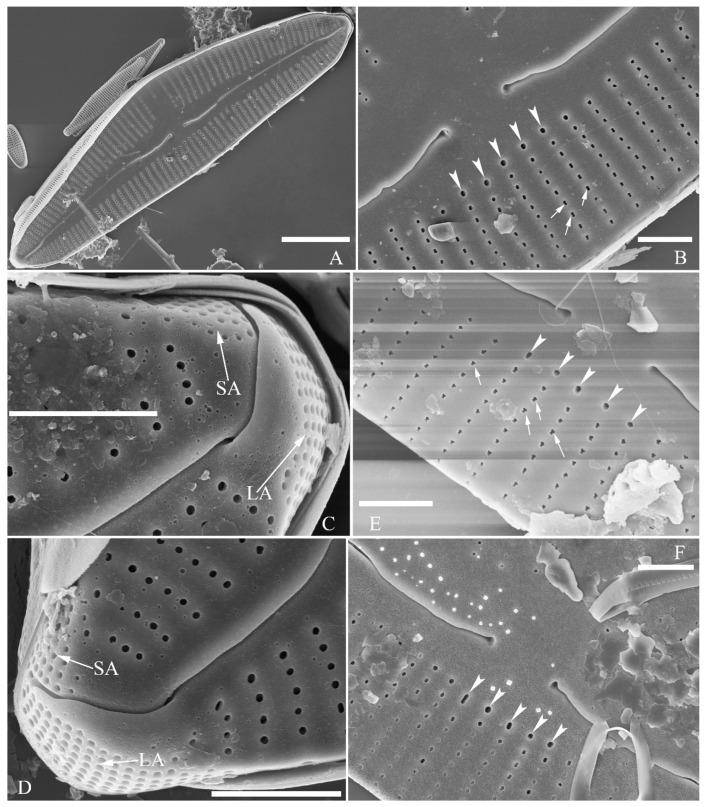
*Cymbella apiculatophora* sp. nov., SEM, valve external view. (**A**). A frustule. (**B**). Middle part details; note ca. 5 stigmata (five arrowheads) and rounded or dumbbell-like (three arrows) outer openings of areolae. (**C**,**D**). Details of two apices from **A**; note the apical field divided by the distal raphe fissure into two unequal areas—a larger area (LA) and a smaller area (SA)—and the rounded outer openings of areolae near each apex. (**E**,**F**). Two other middle part details; note ca. 5 stigmata (five arrowheads) and the rounded or dumbbell-like (four arrows) outer openings of areolae. Scale bars (**A**) = 10 μm, (**B**–**F**) = 2 μm.

**Figure 3:**
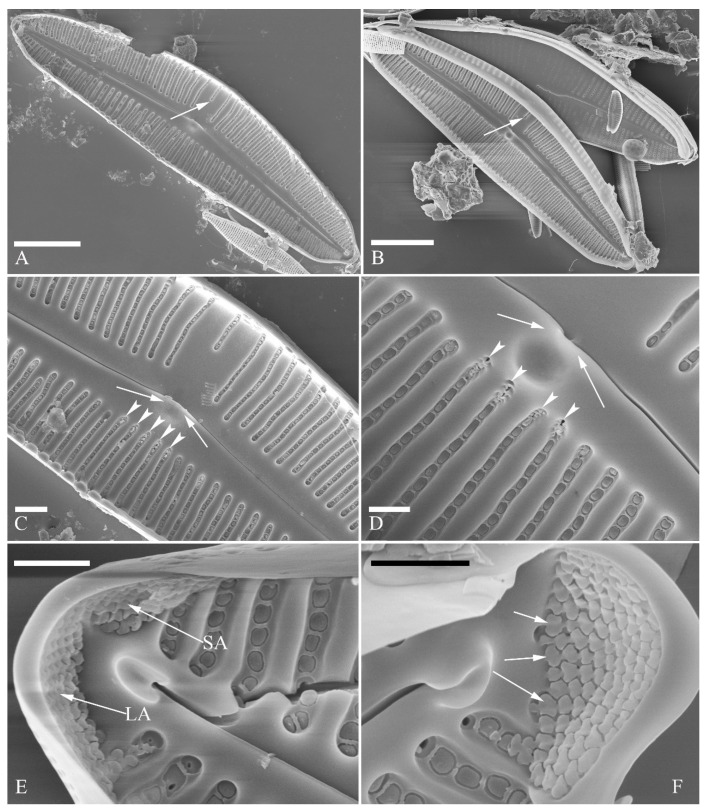
*Cymbella apiculatophora* sp. nov., SEM, valve internal view. (**A**,**B**). Two valves; note a shortened stria on the dorsal middle part (two arrows). (**C**,**D**). Middle part details, note ca. 4 or 5 stigmata (arrowheads) and obscured intermissio (two arrows, respectively). (**E**). Apical detail; note the apical field divided by the distal raphe fissure into two unequal areas: a larger area (LA) and a smaller area (SA). (**F**). Apical detail; note the columns of V-shaped occlusions (three arrows). Scale bars (**A**,**B**) = 10 μm, (**C**) = 2 μm, (**D**–**F**) = 1 μm.

**Figure 4:**
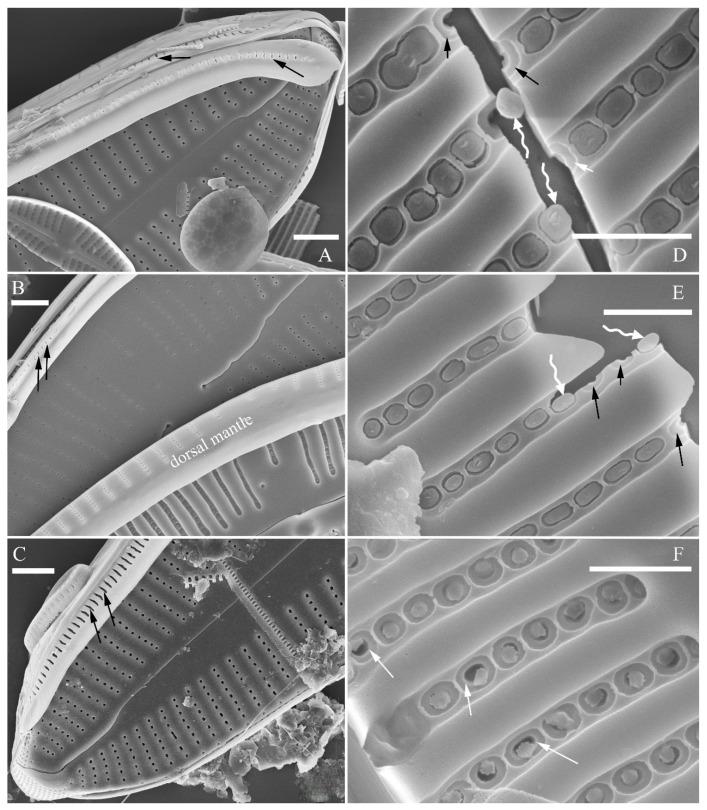
*Cymbella apiculatophora* sp. nov., SEM. (**A**–**C**). Details showing open girdle bands and a row of large, elongated pores located along the midline of copula (two arrows, respectively). (**D**–**F**). Internal details; note manhole-shaped internal openings (arrows) and their rounded to oblong silica closing plates (wavy arrows). Scale bars (**A**–**C**) = 2 μm, (**D**–**F**) = 1 μm.

***Cymbella apiculatophora*** Bing Liu and S. Blanco sp. nov. (Figure 1, Figure 2, Figure 3 and Figure 4).

**Description.** LM (Figure 1). Living cells in valve view have same outlines as valves (Figure 1A,B). Valves moderately dorsiventral with convex dorsal margin and slightly tumid ventral margin. Valve apices apiculate. Valve dimensions (*n* = 23): length 60–66 μm, width 15.5–18.5 μm, length/width ratio range 3.5–4.5. Axial area lanceolate. Central area trapezoid, more developed on dorsal side. Raphe slightly lateral, becoming filiform near distal and proximal ends. Central pores visible, bulbous. Striae radiate throughout valve surface. One shortened stria, widely spaced from each adjacent stria, less than half length of adjacent stria, always present on dorsal middle part of valve (e.g., Figure 1C–F, arrows; see also Figure 2A and Figure 3A,B). Striae, 10–12 in 10 μm in ventral middle part of valve. Areolae discernible, 18–24 in 10 μm. Stigmata, four to six, present on ventral side of central nodule, very close to ends of corresponding ventral striae.

SEM, external view (Figure 2 and Figure 4A–C). Proximal raphe endings expanded (Figure 2A,B,E,F), distal raphe fissures deflected towards dorsal side of valve and dividing each apical pore field into two unequal areas: a larger ventral one composed of ca. 14–18 pervalvar columns of rounded porelli (each column comprising ca. 2–8 porelli) and a smaller dorsal one composed of ca. 6–9 pervalvar columns of rounded porelli (each column comprising ca. 2–8 porelli) (Figure 2C,D and Figure 4C). Four to six stigmata located on the ventral side of central nodule with rounded to oblong outer openings (Figure 2B,E,F). Areola openings rounded near apex, similar to porelli of apical pore field (Figure 2C,D) or dumbbell-like (Figure 2B,E, arrows). Girdle bands open with a row of large, elongated pores located along midline of copula (Figure 4A–C, two arrows, respectively).

SEM, internal view (Figure 3 and Figure 4D–F). Raphe straight, almost along valve midline, proximal raphe endings hidden, i.e., intermissio invisible due to being covered by siliceous hood (Figure 3A–D), distal raphe fissures terminating in raised bilabiate helictoglossae (Figure 3E,F). Four to six stigmata located on ventral side of central nodule with convoluted internal occlusions (Figure 3C,D, arrowheads). Structure of areolar inner openings similar to that of manhole covers, i.e., areolar inner openings located in the middle of rounded depression that is completely covered by rounded to oblong solid silica plates (Figure 4D–F). Apical pore fields composed of a larger ventral area and a smaller dorsal area (Figure 3E). Porellus openings of apical pore fields covered by columns of silica strips composed of V-shaped plates (Figure 3F, arrows).

**Holotype designated here**. Slide DIA2024004, specimen circled on the slide, illustrated here as Figure 1I, deposited in the herbarium of Jishou University (JIU), China. Registration: http://phycobank.org/104759.

**Type locality.** China. Hunan Province, Shimen County, Huping Town, Xie River. A specific sampling location (29°57′6″ N, 110°45′37″ E, 230 m a.s.l.) in a riffle of the Xie River, collected by Bing Liu, 14 March 2021.

**Etymology.** The epithet *apiculatophora* refers to the abrupt, short, pointed valve apices of this new species.

**Ecology and distribution.** The samples that included this species were scraped off the surface of stones collected in the Xie River. Hence, this is an epilithic species. The following environmental parameters were measured in the field with three replications: Conductivity = 236.3 ± 1.2 μS∙cm^−1^; pH = 8.49 ± 0.02; water temperature = 13.6 ± 0.1 °C. Known only from the type locality so far.

**Comments.** *Cymbella apiculatophora* sp. nov. is characterized by its dorsiventral valve outline, one shortened stria located on the dorsal middle part of valve, a large dorsal central area and its apiculate apices. The most similar species to *C. apiculatophora* is *C. neuquina* Frengueli and its variety *C. neuquina* var. *fastigata* (Krasske) Krammer, Maidana & Villanueva. All three taxa have similar valve outlines and apices, but *C. apiculatophora* bears one distinctly shortened stria on the dorsal middle part, whereas *C. neuquina* and the variety *fastigata* do not have this character (Table 3). The morphometric data, such as stria and areola densities, are also noticeably different (see Table 3). Four low-resolution SEM images for *C. neuquina* were provided in Maidana et al. [19]. From their figure 23, we can see that *C. neuquina* has an APF composed of a complete area, thus differing from that composed of two unequal areas in *C. apiculatophora* (Table 3). *Cymbella orientalis* and its variety *C. orientalis* var. *delicatula* Stancheva & Ivanov also have a large dorsal central area, but these two taxa differ from *C. apiculatophora* by their weakly dorsiventral valve outline, narrowly rounded apices and lack of a stigma (Table 3). Interestingly, the apical pore fields of *C. apiculatophora* and *C. orientalis* are very similar in structure and their areola internal openings are completely covered by solid closing plates. For both species, the porelli have similar size and shape as the areolae, such that they could be classified as undifferentiated [20], i.e., their APFs are not clearly physically separated and morphologically differentiated from the striae.

**Figure 5:**
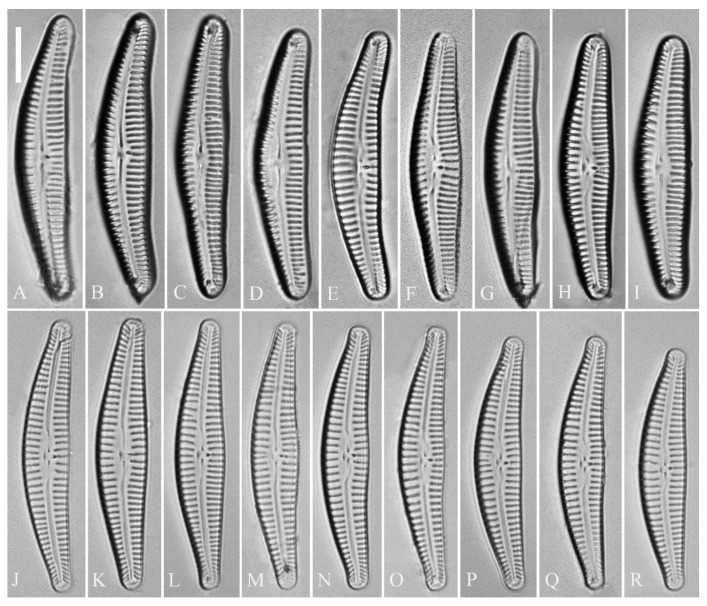
*Cymbella* cf. *excisiformis*, LM. (**A**–**I**). Nine pre-normal valves; note that their somewhat vaulted outline. (**J**–**R**). Nine normal vegetative valves. Scale bar (**A**–**R**) = 10 μm.

**Description.** LM (Figure 5). Pre-normal valves somewhat vaulted (Figure 5A–I). Normal valves dorsiventral, dorsal margin strongly convex, ventral margin slightly convex. Apices subrostrate to subcapitate. Valve dimensions (*n* = 36): length 44–53 μm, width 8–9.5 μm. Axial area narrow. Central area present only in pre-normal valves (Figure 1A–I), in normal valves nearly absent (Figure 1J–R). Raphe lateral, slightly reverse-lateral towards valve central part. Striae slightly radiate in middle of valve, radiate towards apices. Areolae difficult to discern under LM. An isolated stigma located on ventral side of central nodule. Striae in dorsal middle part, 8–10 in 10 μm; in ventral middle part, 9–12 in 10 μm. Puncta, 28–32 in 10 μm.

**Figure 6:**
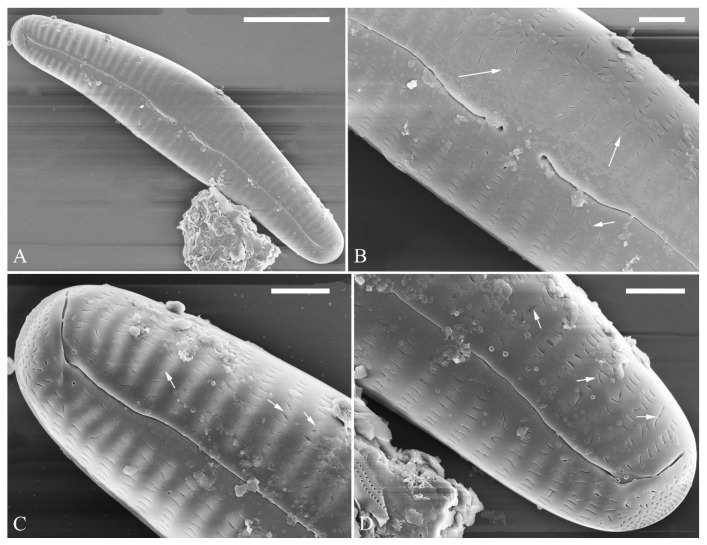
*Cymbella* cf. *excisiformis*, pre-normal valve, SEM, external view. (**A**). A complete pre-normal valve. (**B**–**D**). Details from **A**. Note lineolate areola openings; most of them are oriented parallel to the apical axis, but some are oriented transapically or at an angle relative to the apical axis (arrows). Scale bars (**A**) = 10 μm, (**B**–**D**) = 2 μm.

**Figure 7:**
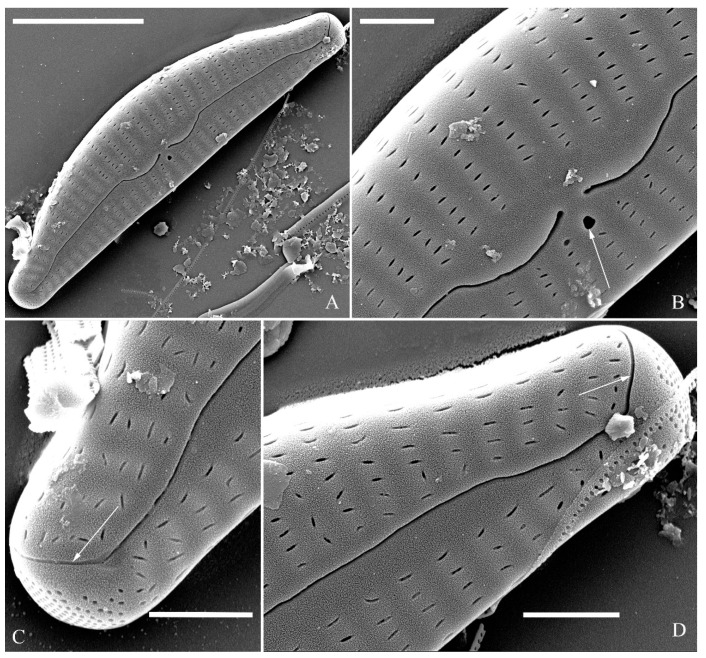
*Cymbella* cf. *excisiformis*, SEM, external view. (**A**). A complete valve; note the lateral-reverse proximal raphe fissures. (**B**). Detail of middle part; note the stigma (arrow). (**C**,**D**). Two apical details; note that the distal raphe fissure does divide the apical pore field into two areas (arrows). Scale bars (**A**) = 10 μm, (**B**–**D**) = 2 μm.

SEM, external view (Figure 6 and Figure 7). Pre-normal valves more vaulted and having lineolate areola openings oriented more transapically or at an angle relative to apical axis than in normal vegetative valves (Figure 6A–D). Proximal raphe fissures reverse-lateral (Figure 7A,B), dorsal raphe fissures deflected towards dorsal side (Figure 7C,D). External opening of stigma rounded (Figure 7B, arrow). Most areola openings lineolate, apically oriented; some not. Apical pore fields composed of a single area, not divided by distal raphe into two unequal areas (Figure 7C,D, arrows, respectively).

**Figure 8:**
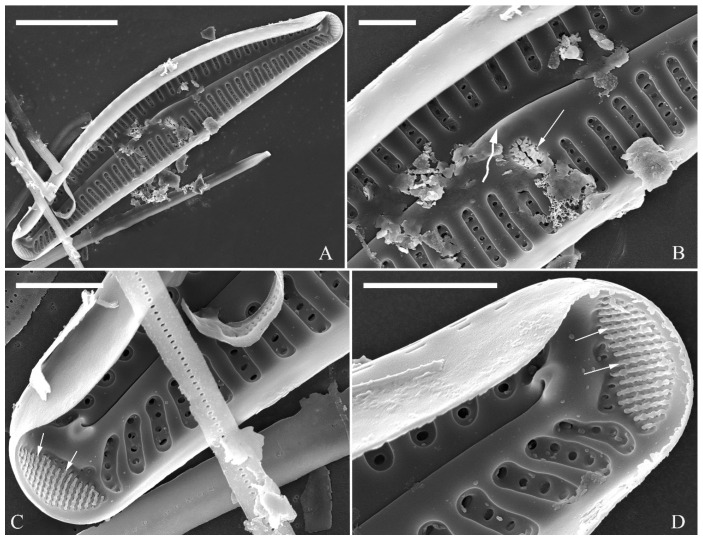
*Cymbella* cf. *excisiformis*, SEM, internal view. (**A**). A complete valve. (**B**). Detail of middle part; note the stigma (arrow) and obscured intermissio (wavy arrow). (**C**,**D**). Two apical details; note an undulate flap-like silica strip covering the internal apertures of each column of foramina but not occluding the apertures completely (two arrows, respectively). Scale bars (**A**) = 10 μm, (**B**–**D**) = 2 μm.

SEM, internal view (Figure 8). Proximal raphe endings obscured by a silica hood so that the intermissio is invisible (Figure 8A,B, wavy arrow). Distal raphe fissures terminating in raised, bilabiate helictoglossae. Internal opening of stigma with convoluted occlusions (Figure 8B, arrow). Internal areola openings located in shallow depressions between two adjacent virgae, rounded, no occlusion present. APFs composed of a single area. An undulate silica strip covering each column of foramina but not completely occluding (Figure 8C,D, two arrows, respectively).

**Comments.** *Cymbella* cf. *excisiformis* was commonly found with *C. menyuanensis* sp. nov. in an unnamed river (37°27′28″ N, 101°23′15″ E, 2940 m a.s.l.) in Menyuan County, Qinghai Province, China. It lives on the stone surfaces of a plateau river. In the original description of *C. excisiformis*, Krammer [1] reported that its valve length range is 18–44 μm and its density of puncta is 24–30 in 10 μm. Our population has larger cells than Krammer’s (44–53 vs. 18–44 μm) and has a higher density of areolae (28–32 vs. 24–30 in 10 μm). In our population we found an initial valve and many pre-normal valves but did not find specimens smaller than 44 µm in length. Our population is similar to the larger specimens of *C. excisiformis* illustrated by Krammer [1]; therefore, we identified it as *C.* cf. *excisiformis*. Below, we will describe in detail its initial valve.

**Figure 9:**
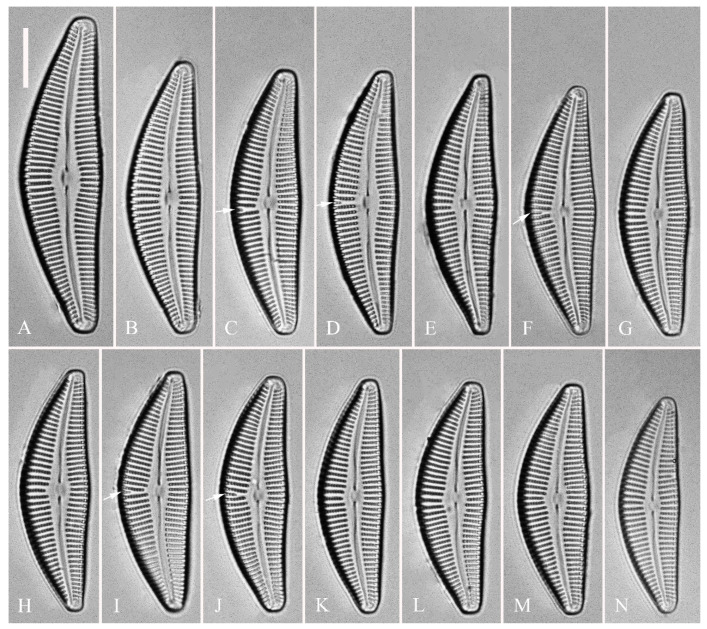
*Cymbella hunanensis*, sp. nov., LM. (**A**–**N**). Fourteen valves in a series of diminishing size; note that a shortened stria is sometimes present on the dorsal central part (arrows). (**A**). Illustration of the holotype specimen. Scale bar (**A**–**N**) = 10 μm.

**Figure 10:**
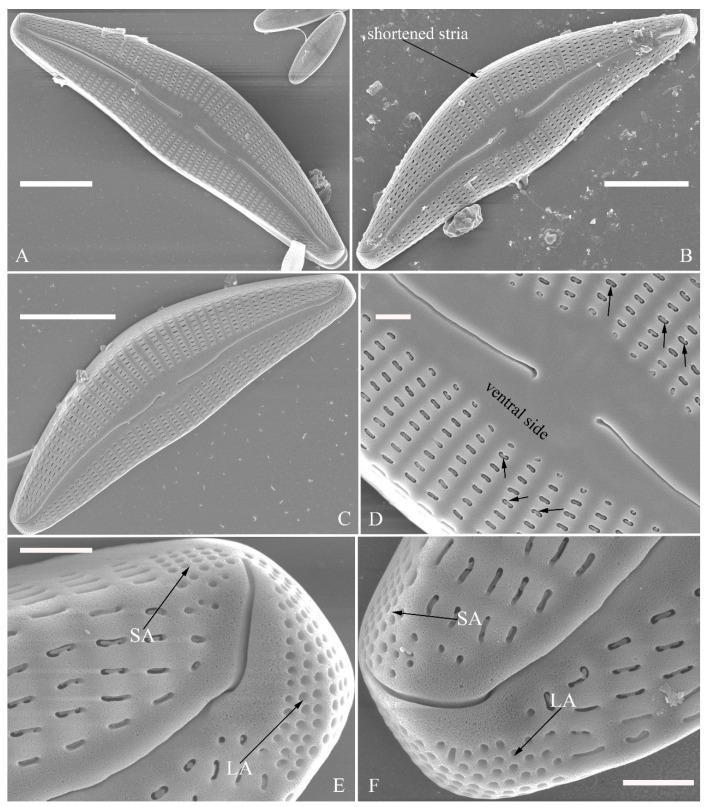
*Cymbella hunanensis*, sp. nov., SEM, external view. (**A**–**C**). Three valves; note that a shortened stria is sometimes present on the dorsal central part (arrow in **B**). (**D**). Middle part details; note the reniform closing plates (black arrows) and absence of a stigma. (**E**,**F**). Details of two apices from **C**. Note the apical field divided by the distal raphe fissure into two areas: a larger area (LA) and a small area (SA). Scale bars (**A**–**C**) = 10 μm, (**D**–**F**) = 1 μm.

**Figure 11:**
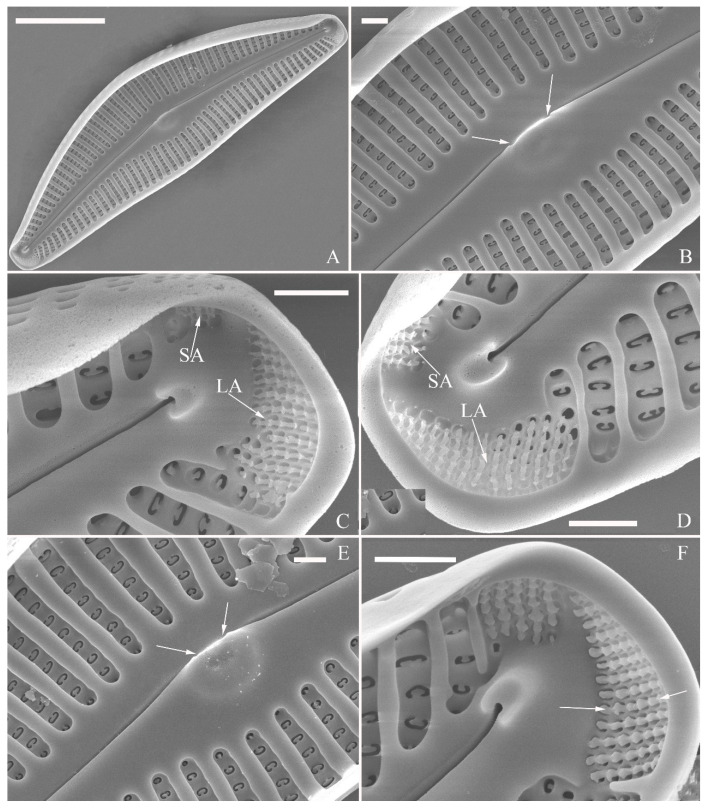
*Cymbella hunanensis*, sp. nov., SEM, internal view. (**A**). A complete valve. (**B**). Middle part details; note the two internal proximal raphe endings interrupted by the central nodule (two arrows) and the absence of stigmata. (**C**,**D**). Apical details. Note the apical field divided into two areas: a large area (LA) and a small area (SA). (**E**). Other middle part details; note the two internal proximal raphe endings interrupted by the central nodule (two arrows) and the absence of stigmata (arrows). (**F**). Details showing an undulate flap-like silica strip above the internal apertures of each column of foramina but not occluding the internal apertures completely (two arrows in **F**). Scale bars (**A**) = 10 μm, (**B**–**F**) = 1 μm.

***Cymbella hunanensis*** Bing Liu & Rioual sp. nov. (Figure 9, Figure 10 and Figure 11).

**Description.** LM (Figure 9). Valves slightly dorsiventral, almost rhombic–lanceolate, dorsal margin highly arched, ventral margin slightly convex due to presence of a slightly gibbous central portion. Valve apices cuneate, obtuse, not protracted. Valve dimensions (*n* = 42): length 32–56 μm, width 8.5–12.5 μm. Axial area lanceolate. Central area elliptical. Raphe slightly lateral, proximal raphe fissures almost straight with small central pores. Stigmata absent. Striae radiate throughout valve surface, 10–12 in 10 μm in dorsal middle part, 11–13 in 10 μm in ventral middle part. A shortened stria sometimes produced on dorsal middle part (Figure 9C,D,F,I,J, arrows, respectively). Areolae discernible, 20–25 in 10 μm.

SEM, external view (Figure 10). Proximal raphe endings expanded (Figure 10A–D), distal raphe fissures dorsally deflected, divided apical pore fields into two unequal areas: a larger ventral area (LA) composed of ca. 14–18 pervalvar columns of porelli (each column composed of ca. 1–7 porelli) and a smaller dorsal area (SA) composed of 4–7 pervalvar columns of porelli (each column composed of ca. 1–7 porelli) (Figure 10E,F). Areola outer openings reniform; areolar occlusions (closing plates) also reniform, with strut affixed to areolar wall, produced below valve surface, partially occluding areolae (Figure 10D, arrows).

SEM, internal view (Figure 11). Raphe straight, proximal raphe endings interrupted by central nodule, intermissio clearly visible (*ca.* 1.5 μm long), i.e., no silica hood obscuring intermissio (Figure 11A,B,E), distal raphe fissure terminating in raised bilabiate helictoglossa (Figure 11C,F). Areolae’s internal openings oblong, located in depression between two adjacent virgae, occluded by reniform closing plates. APFs composed of a larger ventral area and a smaller dorsal area (Figure 11C,F). An undulate flap-like silica strip covering apertures of each pervalvar column of porelli but not completely occluding them (Figure 11F, two arrows).

**Holotype**. Slide DIA2024005, specimen circled on the slide, illustrated here as Figure 9A, deposited in the herbarium of Jishou University (JIU), China.

Registration: http://phycobank.org/104760.

**Type locality.** China. Hunan Province, Yuanling County, Shenxi River. A specific sampling location (28°44′48″ N, 110°25′27″ E, 200 m asl.) in a riffle of the Shenxi River, collected by Bing Liu, 17 March 2017.

**Etymology.** The epithet *hunanensis* is derived from Hunan Province, where this new species was found.

**Ecology and distribution.** The diatom samples were scraped off of stone surfaces. Hence, this is an epilithic species. The following environmental parameters were measured in the field**.** pH = 8.3 ± 0.1, conductivity = 215.7 ± 2.6 μS∙cm^−1^, water temperature = 12.2 ± 0.1 °C. Known from the type locality and the Li River, Sangzhi County, Hunan Province, China.

**Comments.** *Cymbella hunanensis* sp. nov. is characterized by its slightly dorsiventral and almost rhombic-lanceolate valve outline, lack of stigma, clearly visible intermissio, areolae occluded by reniform closing plates and apical pore fields divided by the distal raphe fissure into two unequal areas. The two most similar species to *C. hunanensis* are *C. stigmaphora* and *C. subleptoceros* (Table 4). However, unlike these two species, which do not possess a central area, *C. hunanensis* has an elliptical central area (Table 4). Moreover, the apices of *C. hunanensis* are more obtuse than those of *C. stigmaphora* and *C. subleptoceros*.

**Figure 12:**
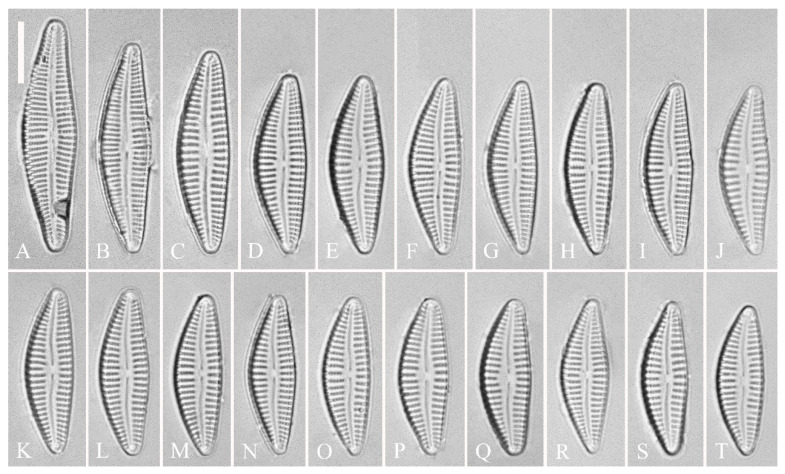
*Cymbella hustedtii*, LM. (**A**–**T**). Twenty valves in a series of diminishing size. Scale bar (**A**–**T**) = 10 μm.

**Description.** LM (Figure 12). Valves slightly dorsiventral, both valve margins convex, but dorsal margin markedly more arched than ventral one. Valve apices cuneate to acute. Valve dimensions (*n* = 45): length 23–37 μm, breadth 5.5–7.5 μm. Axial area narrow; central area absent or, in some specimens, not well expressed on dorsal side. Raphe nearly along valve midline except approaching valve center, where raphe is ventrally displaced. Central pores absent. Striae slightly radiate in middle part, radiate towards apices. Striae in dorsal middle part, 10–14 in 10 μm; in ventral middle part, 10–12 in 10 μm. Stigmata absent. Areolae difficult to discern under LM, 25–31 in 10 μm.

**Figure 13:**
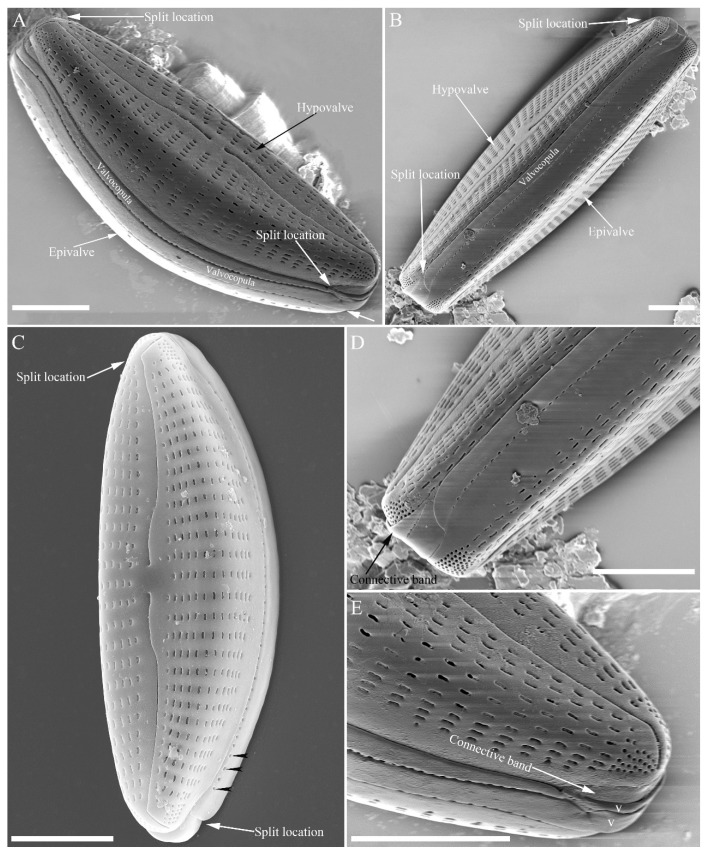
*Cymbella hustedtii*, SEM, external view. (**A**,**B**). Two frustules; note girdle bands and split locations in girdle view. (**C**) Valve with valvocopula; note row of poroids along suture (three black arrowheads). (**D**,**E**). Two apices; note that connective band surrounds insertion of each apex and that two valvocopulae split near apex (**E**, two vs; v = valvocopula). Scale bar (**A**–**E**) = 5 μm.

**Figure 14:**
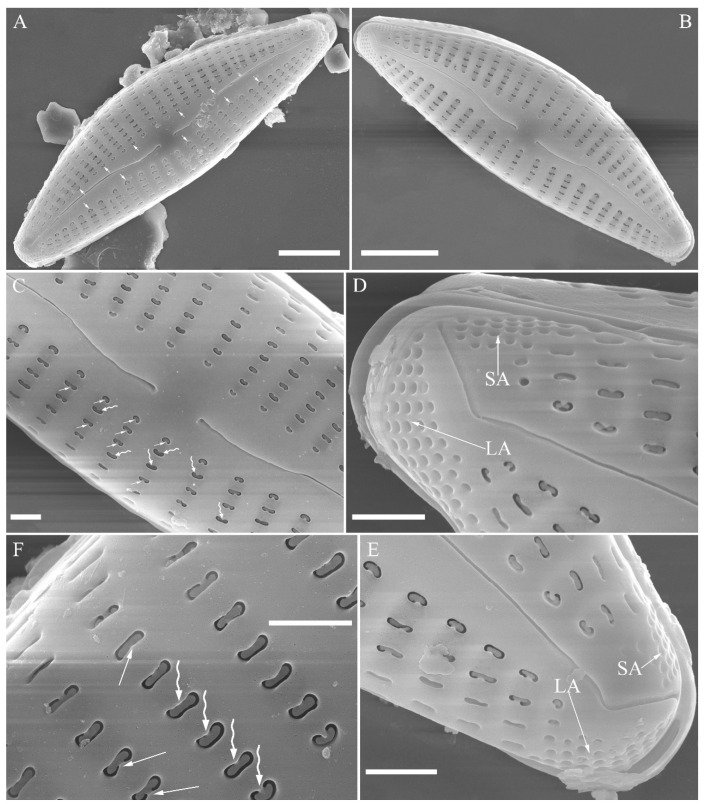
*Cymbella hustedtii*, SEM, external view. (**A**,**B**). Two valves; note that the areolae close to the axis are smaller than the other areolae (**A**, arrows). (**C**,**F**). Details showing reniform external openings of areolae and reniform closing plates. Note the struts attached to the areola lumens on either the dorsal or ventral side (arrows and wavy arrows, respectively). (**D**,**E**). Two apical details. Note the apical field divided by the distal raphe fissure into two unequal areas: a larger ventral area (LA) and a smaller dorsal area (SA). Scale bars (**A**,**B**) = 5 μm, (**C**–**F**) = 1 μm.

SEM, external view (Figure 13 and Figure 14). Frustule with deeper dorsal mantle than ventral mantle (Figure 13A,B). Epicingulum comprising a single valvocopula composed of two parts: one part inserting dorsal mantle margin (Figure 13A), another part inserting ventral mantle margin (Figure 13B). Hypocingulum comprising valvocopula and two connective bands. Valvocopulae of hypocingulum and epicingulum identical in structure and position. Connective bands short, surrounding and inserting each apex of hypovalve (Figure 13D,E, arrows, respectively). For each frustule, there are two split locations for all girdle bands, each split location situated near apex (Figure 13A–C, labelled split location). A row of rounded to oblong poroids dividing valvocopula into pars exterior and pars interior (Figure 13C, three black arrowheads). Proximal raphe endings slightly displaced towards ventral side, distal raphe fissures dorsally deflected, dividing apical pore fields into two unequal areas: a larger ventral area (LA) composed of ca. 9–12 pervalvar columns of porelli (each column composed of ca. 1–5 porelli) and a smaller dorsal area (SA) composed of 4–7 pervalvar columns of porelli (each column composed of ca. 1–5 porelli) (Figure 14A,B,D,E). Areola outer openings reniform, areolae close to axial area smaller than most other areolae (Figure 14A, arrows). Areola occlusions (closing plates) also reniform, developed from strut affixed to areola wall on either its dorsal or ventral side (Figure 14C,D, arrows and wavy arrows, respectively). Reniform closing plates projecting in areola lumens, partially occluding each areolar opening below valve surface (Figure 14F).

**Figure 15:**
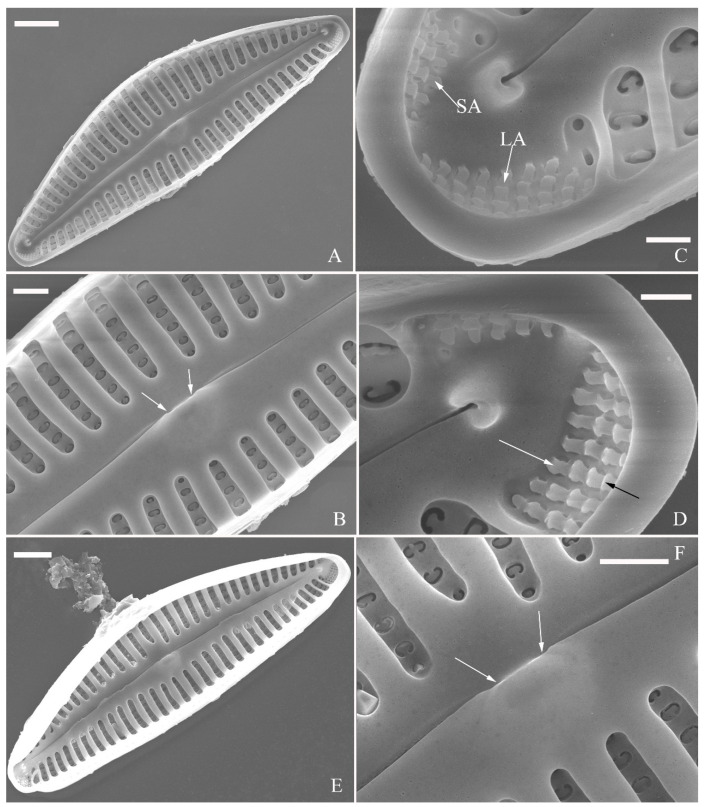
*Cymbella hustedtii*, SEM, internal view. (**A**,**E**). Two complete valves. (**B**,**F**). Middle part details; note that the intermissio is clearly visible (not obscured by a silica hood; two arrows, respectively). (**C**,**D**). Two apical details; note the apical field composed of two areas—a larger area (LA) and a smaller area (SA)—and an undulate flap-like silica strip covering the internal apertures of each column of foramina but not occluding the internal apertures completely (two arrows in **D**). Scale bars (**A**,**E**) = 3 μm, (**B**,**F**) = 1 μm, (**C**,**D**) = 400 nm.

SEM, internal view (Figure 15). Proximal raphe endings interrupted by central nodule, intermissio clearly visible (ca. 1 μm long), not hidden by a silica hood (Figure 15A,B,E,F). Areolae’s internal openings oblong, located in depression between two adjacent virgae, occluded by reniform closing plates.

Distal raphe fissures terminating in raised bilabiate helictoglossae. Apical pore fields composed a larger area and a smaller area (Figure 15C,D). An undulate flap-like silica strip covering internal apertures of each column of porelli but not completely occluding them (Figure 15D, two arrows).

**Comments.** *Cymbella hustedtii* was commonly found with *C. hunanensis* sp. nov. (see above). *Cymbella hustedtii* differs from most *Cymbella* species in its divided APFs, clearly visible intermissio and reniform areola outer openings and closing plates. Five *Cymbella* species have this combination of characters: *C. bourrellyi* Maillard ex Moser, Steindorf & Lange-Bertalot [18], *C. cognata* [5], *C. hustedtii* Krasske [1], *C. subleptoceros* Krammer [1] and *C. hunanensis* sp. nov. (see above). The type species, *C. cymbiformis*, shares dorsal deflected distal raphe fissures, APFs and internal areola occlusions with *C. hustedtii*. The intermissio in *C. cymbiformis* is hidden by a silica hood whereas the intermissio in *C. hustedtii* is clearly visible. These characteristics make C. *hustedtii* an interesting species. Its transfer to the genus *Cymbopleura* by Novelo et al. [21] is, in our opinion, unjustified. On the other hand, Liu et al. [22] did not mention this species when they described the genus *Qinia* Y. Liu, Kociolek & Kulikovskiy, as they only focused their discussion at the generic level.

**Figure 16:**
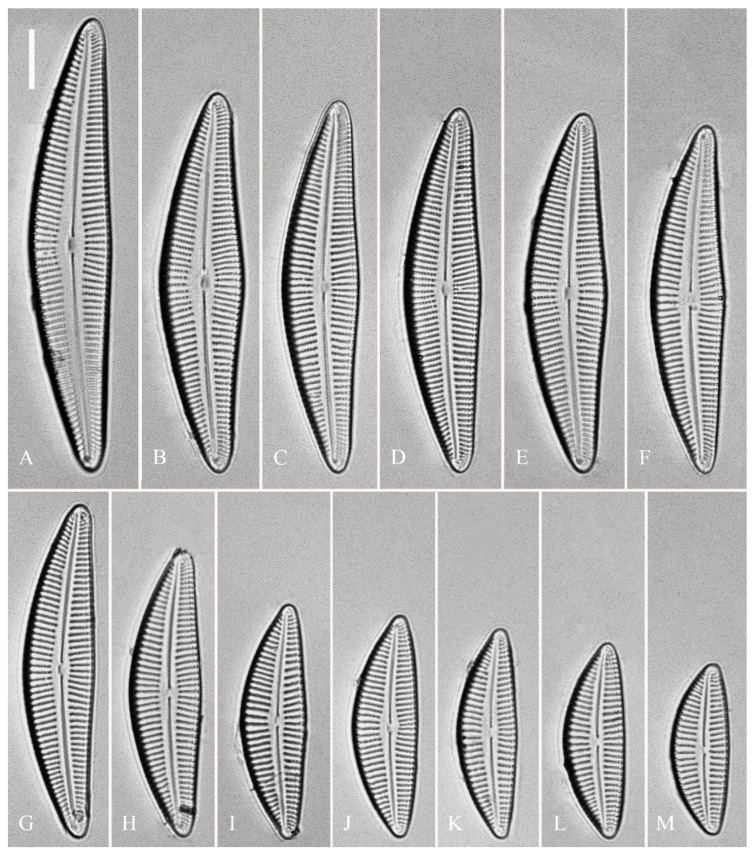
*Cymbella juglandis*, sp. nov., LM. (**A**–**M**). Thirteen valves in a series of diminishing size; note that the ventral margin becomes straighter and straighter in smaller specimens. (**A**). Illustration of the holotype specimen. Scale bars (**A**–**M**) = 10 μm.

**Figure 17:**
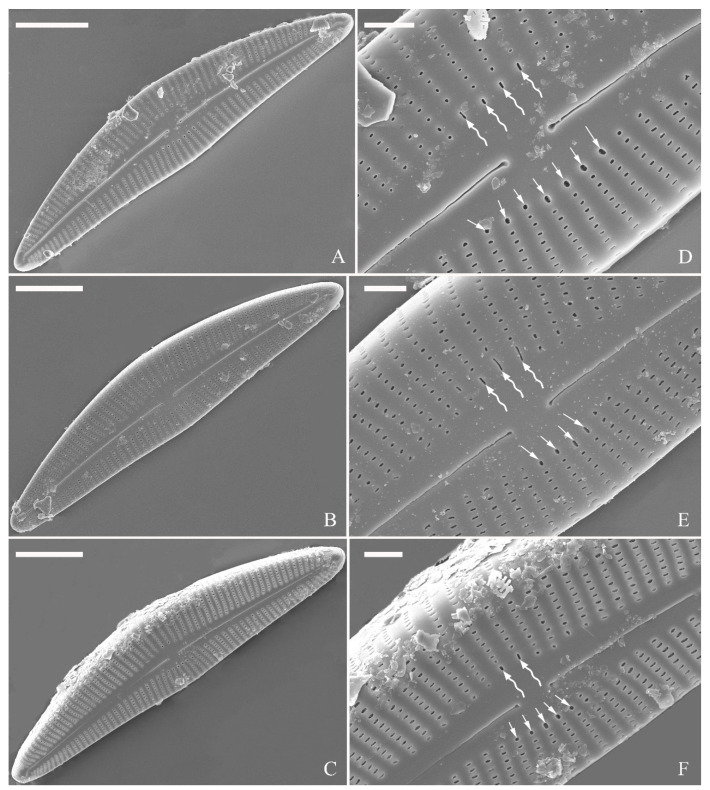
*Cymbella juglandis*, sp. nov., SEM, external view. (**A**–**C**). Three valves; note the almost straight raphes and the acute apices. (**D**–**F**). Middle part details; note that ca. 2–4 terminal areolae in the dorsal central part have different shapes from the others (wavy arrows) and that ca. 4–7 terminal areolae in the ventral central part also have different shapes from the others (arrows). Scale bars (**A**–**C**) = 10 μm, (**D**–**F**) = 2 μm.

**Figure 18:**
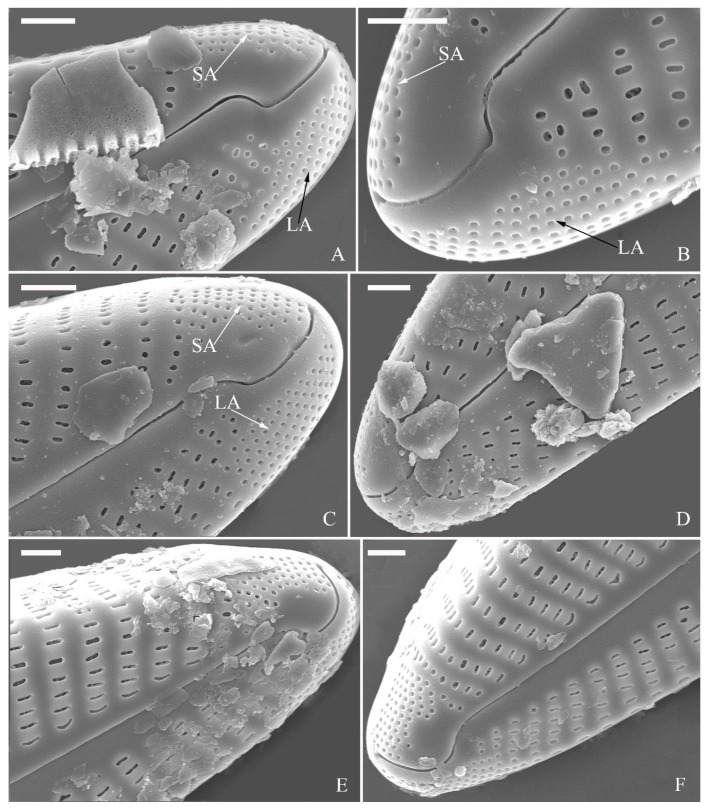
*Cymbella juglandis*, sp. nov., SEM, external view. (**A**–**F**). Apical details. Note the apical field divided by the distal raphe fissure into two areas: a larger area on the ventral side (LA) and a small area on the dorsal side (SA). Scale bars (**A**–**F**) = 1 μm.

**Figure 19:**
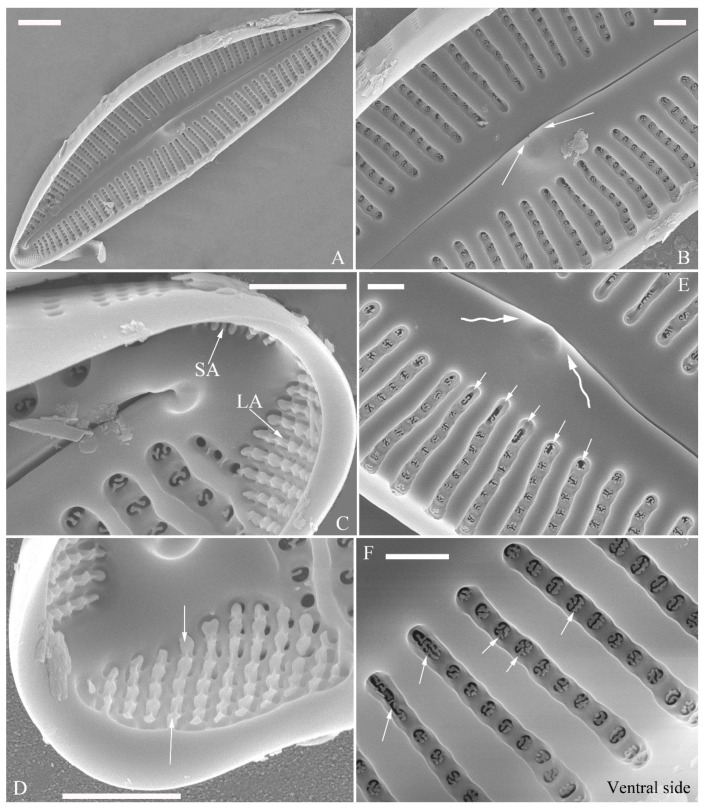
*Cymbella juglandis*, sp. nov., SEM, internal view. (**A**). A complete valve. (**B**). Middle part details; note the obscured intermissio (two arrows) and absence of stigmata. (**C**,**D**). Apical details; note the apical field divided into two areas—a large area (LA) and a small area (SA)—and an undulate flap-like silica strip above internal apertures of each row of foramina but not occluding the internal apertures completely (two arrows in **D**). (**E**). Other middle part details; note the obscured intermissio (two wavy arrows) and absence of stigmata (arrows). (**F**). Details showing the walnut-kernel-like closing plates (arrows). Scale bars (**A**) = 4 μm, (**B**–**F**) = 1 μm.

***Cymbella juglandis*** Bing Liu & S. Blanco sp. nov. (Figure 16, Figure 17, Figure 18 and Figure 19)

**Description. LM** (Figure 16). Valves slightly dorsiventral, almost lanceolate, dorsal margin arched, ventral side slightly convex except in smaller specimens where ventral side almost straight (Figure 16L,M). Valve apices acuminate. Valve dimensions (*n* = 39): length 28–75 μm, width 8–12 μm. Axial area variable, from narrow in smaller specimens to moderately wide in larger specimens, broadening gradually towards valve center. Central area elliptical in larger specimens, indistinct in smaller specimens. Raphe located along midline, straight (filiform). Central pores small. Stigmata absent. Striae radiate throughout valve surface, 10–12 in 10 μm in dorsal middle part, 10–13 in 10 μm in ventral middle part. Areolae discernible under LM, 22–27 in 10 μm.

SEM, external view (Figure 17 and Figure 18). Raphe straight (Figure 17A–C), proximal raphe endings slightly expanded (Figure 17D–F), distal raphe fissures deflected towards dorsal side (Figure 18). External openings of areolae mostly slit-like, some rounded. Particularly, a few of the openings bordering dorsal central area are slit-like, transapically oriented (Figure 17D–F, wavy arrows), while a few bordering ventral central area are rounded, separate from ventral striae (Figure 17D–F, arrows). Distal raphe fissures dividing apical pore fields into two unequal areas: a larger ventral area (LA) composed of ca. 14–16 pervalvar columns of porelli (each column composed of ca. 1–7 porelli) and a smaller dorsal area (SA) composed of 9–11 pervalvar columns of porelli (each column composed of ca. 1–7 porelli) (Figure 18). Occlusions produced below valve surface, partially occluding areolae (Figure 18B,D).

SEM, internal view (Figure 19). Raphe straight, proximal raphe endings obscured by silica hood, i.e., intermissio invisible (Figure 19A,B,E), distal raphe fissures terminating in raised bilabiate helictoglossae. Internal view confirms absence of stigmata (Figure 19B,E,F). Internal openings of areolae located in depressions between adjacent virgae, rounded, elliptical, or oblong; areola occlusions (volae) in the shape of walnut kernels, developed from two or more struts that are affixed to areolar wall (Figure 19F, arrows). Apical pore fields composed of two unequal areas (Figure 19C). An undulate silica strip covering each column of porelli but not completely occluding them (Figure 19D, two arrows).

**Holotype**. Slide DIA2024006, specimen circled on the slide, illustrated here as Figure 16A, deposited in the herbarium of Jishou University (JIU), China. Registration: http://phycobank.org/104761.

**Type locality.** China. Hunan Province, Suining County, Changpu Town, Wu River. A specific sampling location (26°34.59′ N, 110°09.19′ E, 300 m a.s.l.) in a riffle of the Wu River, collected by Bing Liu, 22 March 2021.

**Etymology.** The epithet *juglandis* refers to the areola occlusions, which resemble walnut kernels.

**Ecology and distribution.** Epilithic in a mountain river with oligotrophic waters. The following environmental parameters were measured in the field. Conductivity was 99.7 ± 0.3 μS∙cm^–1^, pH was 7.9 ± 0.1 and water temperature was 13.2 ± 0.2 °C. Known only from the type locality so far.

**Comments.** *Cymbella juglandis* has a unique suite of characters: acuminate apices, divided APFs, lack of stigma and areola inner occlusions (volae) in the shape of walnut kernels. *Cymbella juglandis* differs from *C. shii* by morphometrics such as the valve width (the former having much narrower width than the latter, Table 5). *Cymbella juglandis* is distinguished from *C. subleptoceros* by its obscured intermissio, whereas the latter has a clearly visible intermissio (Table 5).

**Figure 20:**
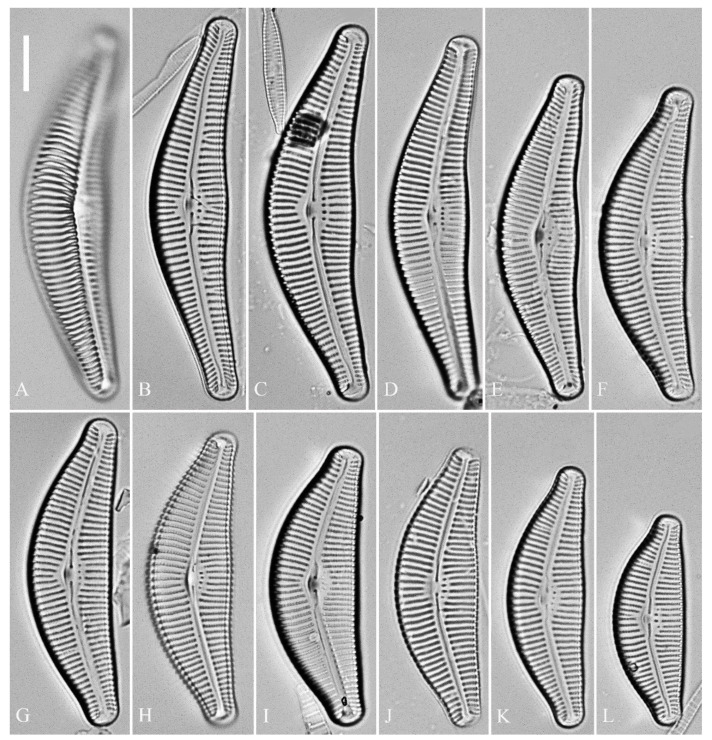
*Cymbella menyuanensis* sp. nov., LM. (**A**). Initial valve or pre-normal valve; note its vaulted outline. (**B**–**L**). Eleven normal vegetative valves in a series of diminishing size. (**B**). Illustration of the holotype specimen. Scale bar (**A**–**L**) = 10 μm.

**Description.** LM (Figure 20). Initial or pre-normal valves vaulted (Figure 20A). Valves strongly dorsiventral, dorsal margin high, strongly arched, ventral margin concave with a swelling in the middle except in small valves which have a straight ventral margin (Figure 20L). Apices rostrate to subcapitate, slightly turned towards dorsal side. Valve dimensions (*n* = 29): length 46–91 μm, width 12.5–20.5 μm. Axial area narrow, linear. Central area small. Raphe lateral, proximal raphe fissures relatively short. Central pores visible. 3–6 stigmata located on ventral side of central nodule, slightly detached from ventral striae. Striae radiate throughout valve surface, 8–11 in 10 μm in both dorsal and ventral middle parts. Areolae discernible under LM, 22–26 in 10 μm.

**Figure 21:**
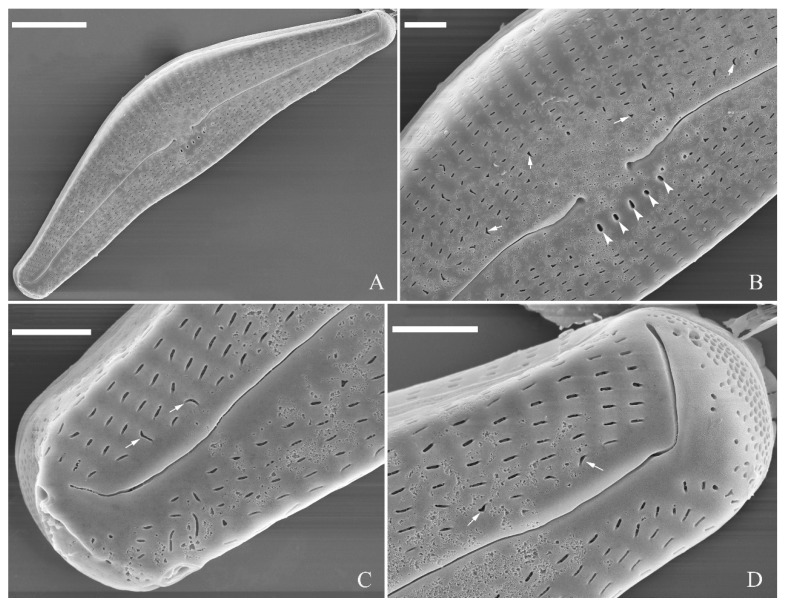
*Cymbella menyuanensis* sp. nov., pre-normal valve, SEM, external view. (**A**). Pre-normal valve. (**B**–**D**). Details from (**A**); note stigmata (arrowheads), various areola openings and orientations (arrows) and poorly developed apical pore fields. Scale bars (**A**) = 10 μm, (**B**–**D**) = 2 μm.

**Figure 22:**
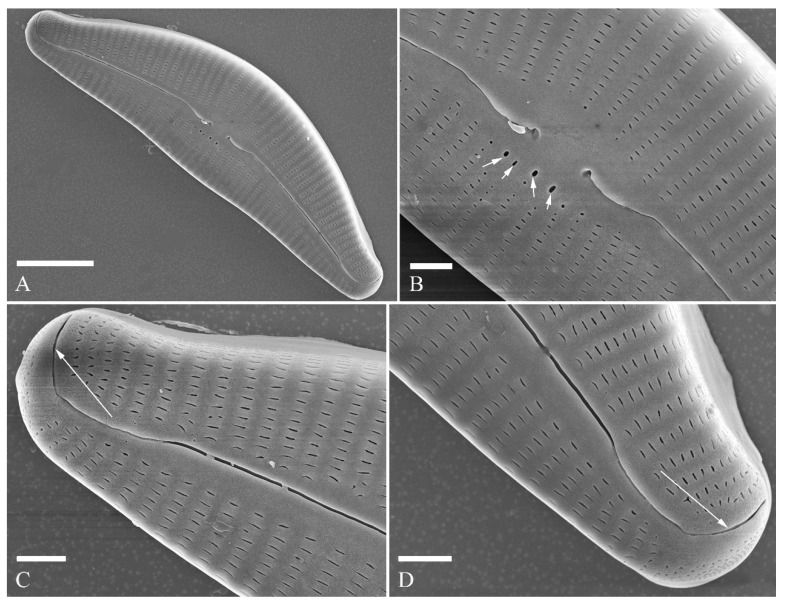
*Cymbella menyuanensis* sp. nov., SEM, external view. (**A**). Normal valve. (**B**). Detail of middle part, note stigmata (arrows). (**C**,**D**). Two apical details, note that the distal raphe fissure does not divide the apical pore field into two areas (two arrows). Scale bars (**A**) = 10 μm, (**B**–**D**) = 2 μm.

SEM, external view (Figure 21 and Figure 22). Areola openings of pre-normal valves have various shapes and orientations (Figure 21, arrows). Apical pore fields not well developed in pre-normal valves (Figure 21C,D). Proximal raphe endings expanded; external openings of stigmata rounded to oblong (Figure 22B). Distal raphe fissures deflected towards dorsal side, not dividing apical pore fields (Figure 22C,D). Most external openings of areolae lineolate (Figure 22B–D).

**Figure 23:**
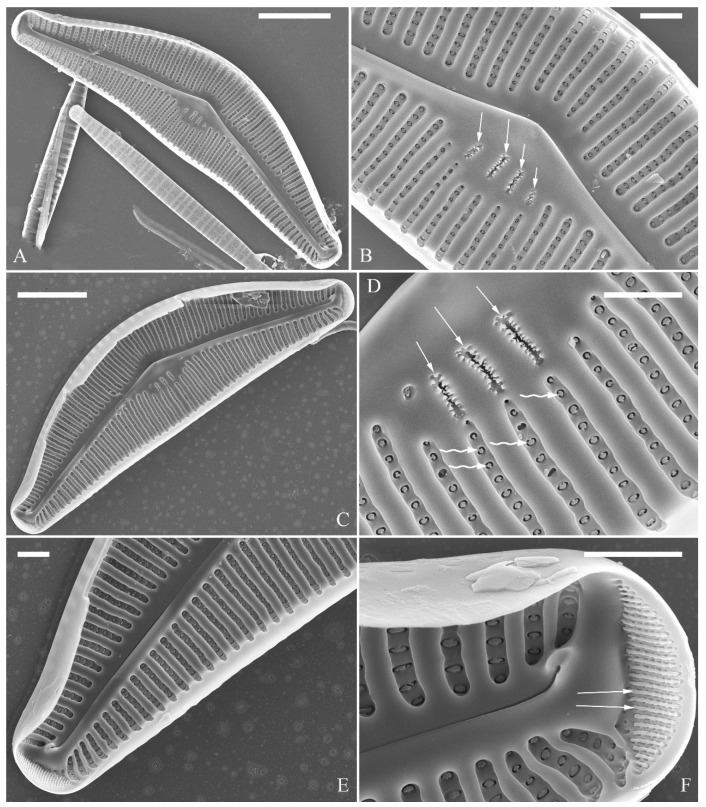
*Cymbella menyuanensis* sp. nov., SEM, internal view. (**A**,**C**). Two complete valves. (**B**,**D**). Details of the middle part; note the stigmata (arrows) and mushroom-shaped closing plates (wavy arrows). (**E**,**F**). Two apical details; note that there is an undulate flap-like silica strip above the internal apertures of each row of foramina but not occluding the internal apertures completely (two arrows). Scale bars (**A**,**C**) = 10 μm, (**B**,**D**–**F**) = 2 μm.

SEM, internal view (Figure 23). Raphe straight, proximal raphe endings obscured by silica hood, i.e., intermissio invisible (Figure 23A–C), distal raphe fissures terminating in raised bilabiate helictoglossae (Figure 23E,F). Internal openings of stigmata with convoluted occlusions (Figure 23B,D, arrows). Internal openings of areolae located in depressions between adjacent virgae, rounded, elliptical or oblong, with mushroom-shaped closing plates developed from strut that is affixed to either dorsal or ventral areolar wall (Figure 23D, wavy arrows). Apical pore fields composed of a single area, an undulate silica strip covering each column of foramina but not completely occluding them (Figure 23F, two arrows).

**Holotype**. Slide DIA2024007, specimen circled on the slide, illustrated here as Figure 20B, deposited in the herbarium of Jishou University (JIU), China.

Registration: http://phycobank.org/104762.

**Type locality.** China. Qinghai Province, Menyuan County, an unnamed river. A specific sampling location (37°27′28″ N, 101°23′15″ E, 2940 m a.s.l.) in a riffle of the unnamed river, collected by Bing Liu, 18 July 2019.

**Etymology.** The epithet *menyuanensis* is derived from Menyuan County of Qinghai Province where this new species was found.

**Ecology and distribution.** The sampling site is located in the plateau which belongs to the highland continental climate zone. The diatom samples were scraped off of the stone surfaces, *Cymbella menyuanensis* is therefore epilithic. The following environmental parameters were measured in the field: Conductivity was 448.3 ± 0.5 μS∙cm^−1^, pH was 8.3 ± 0.1 and water temperature was 11.9 ± 0.5 °C. So far, its distribution is known from the type locality and a river in Huzhu County, Qinghai Province.

**Comments.** *Cymbella menyuanensis* sp. nov. is characterized by its strongly dorsiventral valve outline and rostrate to subcapitate apices, 3–6 stigmata and areolae occluded internally by the mushroom-shaped closing plates. It is similar to *C. neocistula*, *C. nepalensis* and *C. proxima* in morphometry. However, it differs from *C. neocistula* and *C. nepalensis* in its rostrate to subcapitate apices, whereas the latter two have non-protracted, rounded apices (Table 6). It also differs from *C. proxima* in its much higher areola density (22–26 vs. 14–18 in 10 µm, Table 6).

**Figure 24:**
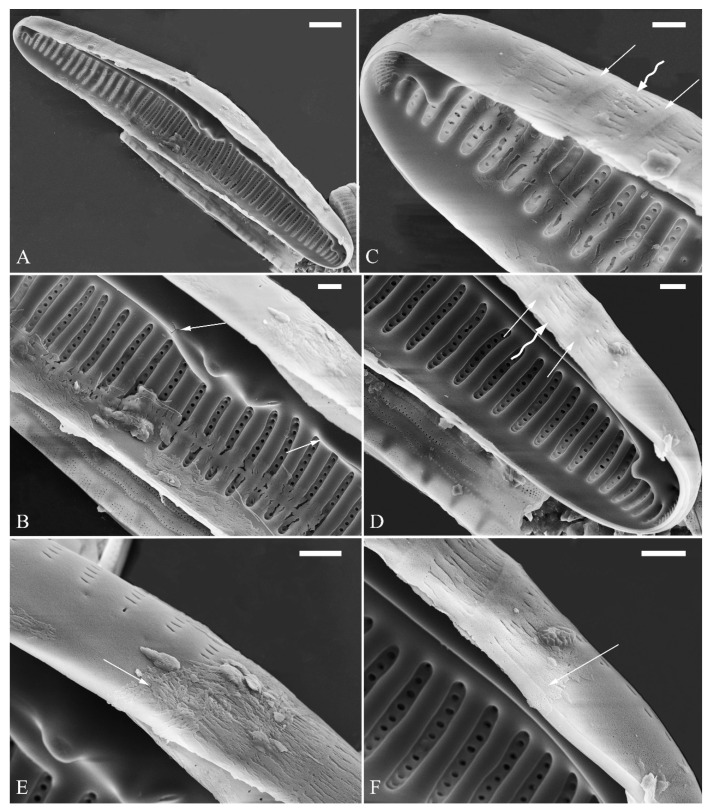
*Cymbella* cf. *excisiformis*, SEM, initial valve. (**A**). An initial valve. (**B**). Detail of the middle part; note the irregular central nodule and two proximal raphe endings (two arrows). (**C**,**D**). Two apical details; note the perizonium composed of a node (two arrows) and internode (wavy arrow). (**E**,**F**). Details showing that the perizonium covers the valve surface before being removed (two arrows). Scale bars (**A**) = 4 μm, (**B**–**F**) = 1 μm.

**Figure 25:**
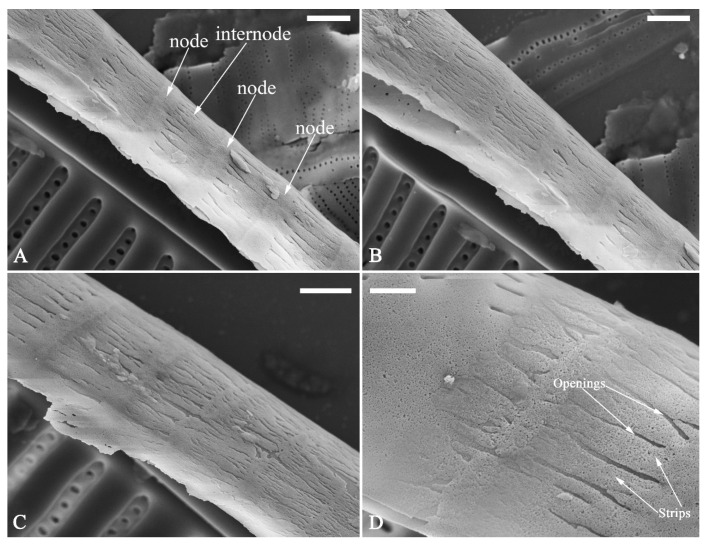
*Cymbella* cf. *excisiformis*, SEM, details of perizonium. (**A**–**D**). The structures of the perizonium, which is composed of two parts: the nodes and the internodes (labeled in **A**). The internode is also composed of two parts: strips and openings between them (labelled in **D**). Scale bars (**A**–**C**) = 1 μm, (**D**) = 400 nm.

**Figure 26:**
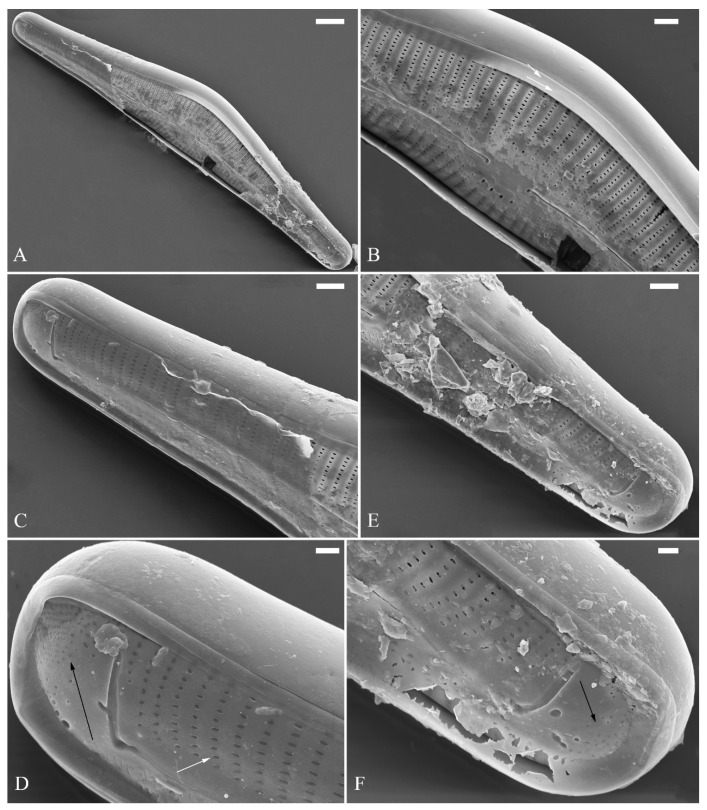
*Cymbella menyuanensis* sp. nov., SEM, initial frustule. (**A**). An initial frustule. (**B**). Detail of the middle part; note the rectangle to oblong external openings of areolae and two girdle bands (two arrows). (**C**,**D**). Two apical details; note that the perizonium encloses the entire frustule, the rectangle to oblong external openings of areolae (white arrow) and irregular apical pore field (black arrow). (**E**,**F**). Details showing that the perizonium covers the valve surface and irregular apical pore field (one black arrow). Scale bars (**A**) = 10 μm, (**B**–**E**) = 3 μm, (**D**,**F**) = 1 μm.

**Figure 27:**
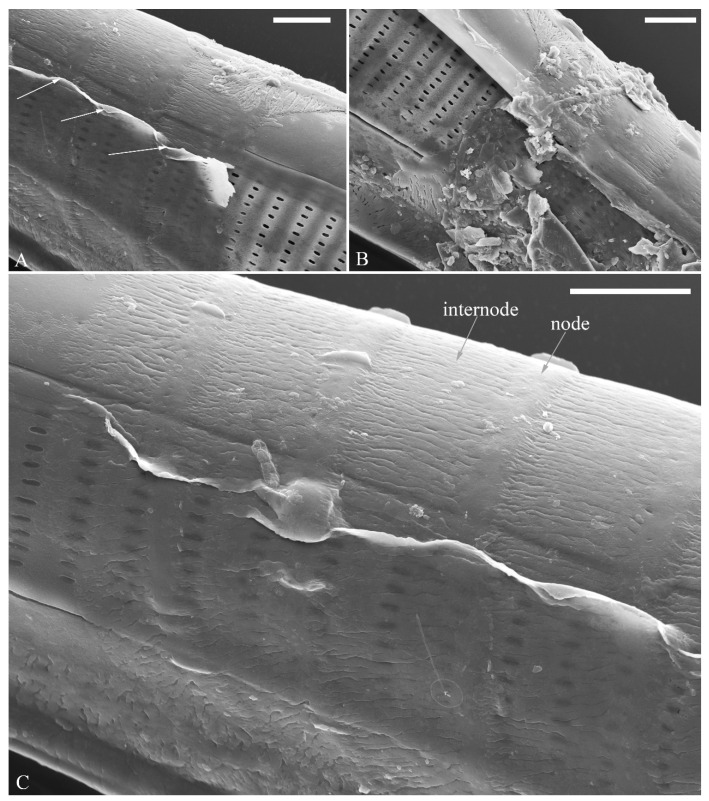
*Cymbella menyuanensis* sp. nov., SEM, details of perizonium. (**A**,**B**). Details showing that the perizonium encloses the entire frustule (three arrows). (**C**). Detail showing that the perizonium is composed of two parts: the nodes and the internodes. The internode is also composed of two parts: strips and openings between them. Scale bars (**A**–**C**) = 3 μm.

A pre-normal valve (Figure 6) and an initial valve (Figure 24 and Figure 25) of *Cymbella* cf. *excisiformis* and an initial frustule (Figure 26 and Figure 27) of *C*. *menyuanensis* sp. nov. were investigated using SEM. The observed initial valve of *C*. cf. *excisiformis* was 52 µm long, 8 µm wide. It had slightly radiate striae, 10 in 10 μm in ventral middle part, and an areola density of 30–32 in 10 μm (Figure 24). The initial frustule of *C. menyuanensis* was 147 µm long, 24 µm wide, with radiate striae, 8 in 10 μm in both the dorsal and ventral middle parts and an areola density of 19–22 in 10 μm (Figure 26).

The perizonia in both *C.* cf. *excisiformis* and *C. menyuanensis* are very similar in structure. They are composed of a whole silica sheet covering the surface of the initial frustule (Figure 24, Figure 25 and Figure 26). The whole perizonium joins (overlaps) at the girdle bands (Figure 26C–F and Figure 27A,C) and is composed of two parts: nodes and internodes (Figure 25 and Figure 27C). The nodes are solid and thickened, resembling the transverse perizonium bands in other diatoms. The internodes are also composed of two parts: strips and openings between them (Figure 25D and Figure 27C). The nodes and internodes are fused together and do not merely overlap with each other. The perizonium is composed of alternate nodes and internodes (Figure 25 and Figure 27C). No transverse perizonium bands were observed.

### 2.2. Discussion

**The perizonium in *Cymbella*.** Kaczmarska et al. [24] defined the perizonium in diatoms as “a part of the auxospore wall comprising silica bands or rings, hoops and strips that is formed underneath the incunabula as the auxospore expands, apparently to control polarity and shape of the growing auxospore and hence also the species-specific shape of the initial cell”. These authors also illustrated two types of perizonia: the transverse and the longitudinal perizonium. Liu and Williams [25] found that *Hannaea inaequidentata* (Lagerstedt) Genkal and Kharitonov only produced the longitudinal perizonium and lacked any transverse perizonium bands. Interestingly, transverse perizonium bands were not found in the two *Cymbella* species investigated in this paper. Liu and Williams [25] defined the pre-normal vegetative period as “the time between immediately after the initial cell’s first division and the presence of the first new normal vegetative cells”. We also found a pre-normal population of *C*. cf. *exciformis* (Figure 5A–I) and illustrated a pre-normal valve (Figure 6). As the ultrastructure of the perizonium in *Cymbella* has never been illustrated in the literature until this study, we hope our findings can generate more interest in investigating this important structure of the life cycle.

**The visible intermissio in *Cymbella*.** Visible intermissiones are seen in a few cymbelloid genera, such as *Cymbopleura* [26,27], *Encyonema* [28,29] and *Vladinikolaevia* [30]. On the contrary, hidden intermissiones have been found in a few cymbelloid genera, such as *Celebesia* [31], *Cymbella* [1,15,32], *Delicatophycus* [3,33], *Karthickia* [34] and *Qinia* [22]. Whether visible intermissio represents a homologous character is unknown. Even in the genus *Cymbella*, at least five species possess a clearly visible intermissio; therefore, whether the intermissio can be used as one of the defining characters to separate a monophyletic group from *Cymbella sensu lato* needs further explorations.

**The divided apical pore fields in *Cymbella.*** Many authors used the term “bisected apical pore fields” for *Cymbella.* This is, however, somewhat misleading, as “bisected” usually refers to a structure divided into two equal parts, which is not the case for *Cymbella* because its distal raphe fissure divides the apical pore fields into two unequal parts. Thus, a more precise terminology for *Cymbella* would require using “divided apical pore fields”. APFs divided by terminal raphe fissures have been observed in other genera within the Cymbellaceae, such as *Cymbellafalsa*, *Celebesia*, *Qinia* or *Reimeria*, and has been a feature typically associated with gomphonemoid diatoms [35]. From our literature survey, we found 16 *Cymbella* species (Table 1) possessing such divided apical pore fields, to which we can add *C. apiculatophora* sp. nov., *C. hunanensis* sp. nov. and *C. juglandis* sp. nov. from the observations presented in this study. Based on the literature for studies with molecular information on *Cymbella* species, there are 18 species of *Cymbella* that have been sequenced [6,27,36,37,38]. Among these 18 species, three have divided APFs and belong to the “aspera group”. A very interesting point is that the “aspera group”, which is composed of *C. aspera, C. himalaspera, C. baicalaspera* and also includes *C. bengalensis*, is well separated from the other *Cymbella* species in all the molecular studies [6,27,36,37,38]. This would suggest that the character “divided APFs” is supported by molecular data. We could not find a SEM image of *C. bengalensis* in the literature (only some LM images in [39]); therefore, we could not confirm if this species also has divided APFs. At this stage, it is unclear if these 19 *Cymbella* species represent a monophyletic group within the Cymbellales. To evaluate this possibility, further explorations, especially molecular analyses, are required.

**Figure 28:**
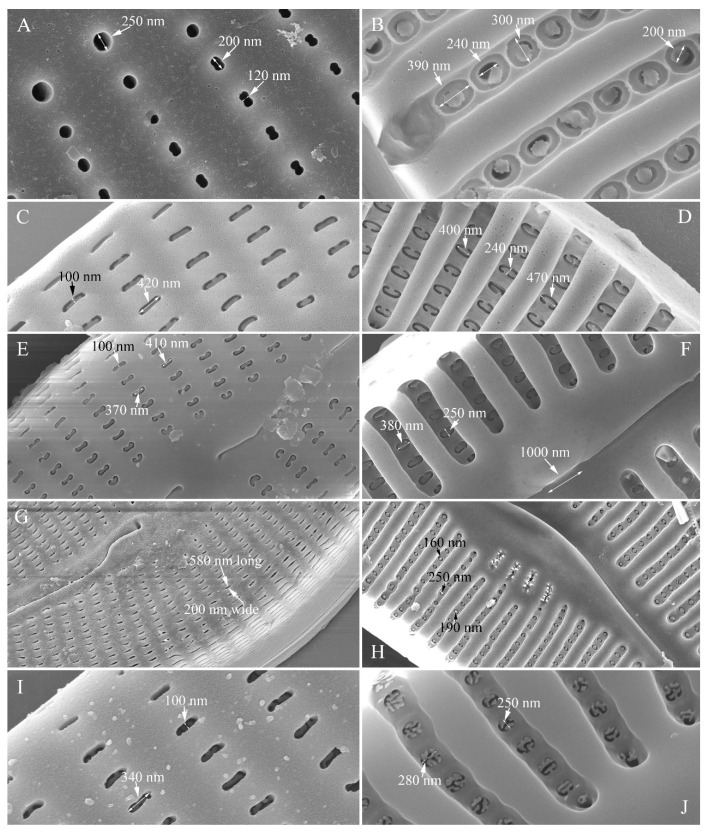
Areolae and their occlusions in five *Cymbella* species. (**A**,**B**). Shapes and sizes of external (**A**) and internal (**B**) openings and occlusions in *C. apiculatophora*. (**C**,**D**). Shapes and sizes of external (**C**) and internal (**D**) openings and occlusions in *C. hunanensis*. (**E**,**F**). Shapes and sizes of external (**E**) and internal (**F**) openings and occlusions in *C. hustedtii*. (**G**,**H**). Shapes and sizes of external (**G**) and internal (**H**) openings and occlusions in *C. menyuanensis*. (**I**,**J**). Shapes and sizes of external (**I**) and internal (**J**) openings and occlusions in *C. juglandis*.

**The various pore occlusions in *Cymbella.*** In this study, the outer openings of areolae are lineolate, and the inner openings of areolae are rounded without occlusions in *Cymbella* cf. *excisiformis*. On the other hand, *Cymbella apiculatophora* sp. nov., *C. hunanensis* sp. nov., *C. hustedtii*, *C. menyuanensis* sp. nov. and *C. juglandis* sp. nov. all produce internal occlusions (Figure 28).

The areola (pore) occlusions were defined in Ross and Sims [40], Ross et al. [41] and Mann [42]. Cox [43] reassessed their structure and terminology and concluded that the closing plate in *Ulnaria* cannot be attributed to a cribrum, hymen, rica, or rota, but it may be considered a vola, which is a catch-all term. To be precise, we use the term closing plate rather than vola. The internal occlusions in *C. hunanensis, C. hustedtii and C. menyuanensis* are solid plates which may be termed as flaps [42] or volae [43]. For this type of structure, the term “closing plates” [44] is more precise. The type of internal occlusions observed in *C. juglandis* appears to be extremely rare in the literature. The shapes and sizes of median-size areolae in the six *Cymbella* species investigated in this study are summarized in Table 7.

**Taxonomic position of the four new species in this paper.** *Cymbella menyuanensis* sp. nov. has a typical cymbelloid valve outline, an obscured intermissio, 3–6 stigmata, internal occlusions of areolae, dorsally deflected distal raphe fissures, and a complete APF at each apex. These features irrefutably support that this taxon is a typical *Cymbella* species belonging to the *C. cymbiformis* Agardh group as defined by Krammer [1]. *Cymbella apiculatophora* has a cymbelloid valve outline, an obscured intermissio, 4–6 stigmata, internal occlusions of areolae, dorsally deflected distal raphe fissures and a divided APF at each apex. These features support a close relationship with the *C. sinensis* Metzeltin & Krammer group according to the phylogenetic hypotheses proposed in Thirouin [35]. *Cymbella hunanensis* sp. nov. has a cymbelloid valve outline, clearly visible intermissio, internal occlusions of areolae, dorsally deflected distal raphe fissures and a divided APF at each apex. These features suggest that it is closely related to the *C. hustedtii* group as defined by Krammer [1]. The last species, *Cymbella juglandis* sp. nov., has a cymbelloid valve outline, an obscured intermissio, internal occlusions of areolae, dorsally deflected distal raphe fissures and a divided APF at each apex. Except for the divided APF at each apex, the features of *C. juglandis* support its classification as a *Cymbella* species. Molecular phylogenetic analyses of the Cymbellales are at the preliminary stage, although the character “divided APFs” is supported by molecular data [6,27,36,37,38]. However, whether the divided APF is a synapomorphy is still unknown. Therefore, at this moment, it is preferable to place *C. juglandis* in the genus *Cymbella*.

## 3. Materials and Methods

The diatom samples of this study were collected from one river in Qinghai and three rivers in Hunan Provinces, China. Qinghai Province occupies the northeast corner of the Qinghai–Tibet plateau, has an average elevation of over 3000 m a.s.l. and has a highland continental climate. Hunan Province is situated in south-central China, which has a subtropical climate. The four rivers sampled are an unnamed river in Menyuan County (37°27′28″ N, 101°23′15″ E, 2940 m a.s.l.), Qinghai Province (*Cymbella* cf. *excisiformis* Krammer and *C. menyuanensis* sp. nov. are described from this river); the Shenxi River in Yuanling County (28°44′48″ N, 110°25′27″ E, 200 m a.s.l.), Hunan Province (*C. hunanensis* sp. nov. and *C. hustedtii* Krasske are described from this river); the Wu River in Suining County (26°34.59′ N, 110°09.19′ E, 300 m a.s.l.), Hunan Province (*C. juglandis* sp. nov. is described from this river); and the Xie River in Shimen County (29°57′6″ N, 110°45′37″ E, 230 m a.s.l.), Hunan Province (*C. apiculatophora* sp. nov. is described from this river). The method of collecting the diatom samples is the same as in Liu [45] and consists of sampling numerous submerged stones showing yellow–brown surfaces that indicate the presence of diatoms. Each stone was placed on a plastic plate, and its surface was brushed using a toothbrush, with the brushed-off diatom samples being washed onto the plate. The diatom samples were transferred into two 100 mL sampling bottles. One bottle was fixed with 70% ethanol, and the other was left unfixed. At the time of sample collection, temperature, pH and conductivity were measured in situ with a portable multimeter (HQ40D, HACH Company).

The laboratory methods are also the same as in Liu [45] and consist of the following: “The collected diatom samples were transported to the lab and used to observe living cells. Part of the samples were fixed with ethanol to a final concentration of ca. 70%. A total of 100 μL diatom samples were transferred into a round chamber (diameter 14 mm, depth 0.35 mm) located in the middle of a custom-made slide by using a pipette, then examined using a Leica DM3000 light microscope (LM), equipped with a Leica MC190 HD digital camera. The collected diatom samples to which 70% alcohol was added were processed (cleaned) for microscopic examination with 10% hydrochloric acid (HCl) and 30% hydrogen peroxide (H_2_O_2_). Permanent slides were prepared using Naphrax mountant and examined using the same light microscope as above. Slides are deposited in the Herbarium of Jishou University, Hunan, People’s Republic of China (JIU) (Herbarium acronyms follow Index Herbarium http://sweetgum.nybg.org/science/ih/). Samples were also examined using scanning electron microscopy (SEM). Several drops of the cleaned diatom material were air-dried on to glass coverslips. The coverslips were attached to aluminium stubs using double-sided conductive carbon strips and sputter-coated with platinum (Cressington Sputter Coater 108auto, Ted Pella, Inc., Redding, CA, USA). Samples were examined and visualised using a field emission scanning electron microscopy (FESEM) Sigma HD (Carl Zeiss Microscopy) available at Huaihua University, China”.

## Figures and Tables

**Table 1:** Sixteen taxa of *Cymbella* having apical pore fields divided by the distal raphe fissure into dorsal and ventral areas.

Taxon	Reference
*C. aspera*	[1] p. 192, plate 1, figure 5; [3] p. 138, figs. 1, 2
*C. cognata*	[5] p. 499, figs. 79, 80
*C. baicalaspera*	[6] p. 5, figs. 6, 7
*C. golestanica (=Qinia golestanica)*	[7] p. 100, figs. 13–15; [8]
*C. himalaspera*	[9] p. 77, figure 3
*C. hustedtii*	[1] p.577, plate 193, figs. 1, 4
*C. latarea*	[10] p. 81, figs. 42, 44
*C. loescherae*	[11] p. 193, figure 15
*C. neoleptoceros*	[1] p. 503, plate 156, figure 5
*C. olgae*	[5] p. 496, figs. 51, 53
*C. orientalis*	[4] p.102, figs. 17, 18, 22
*C. orientalis* var. *delicatula*	[12] p. 460, figs. 16–19
*C. peraspera*	[1] p. 453, plate 131, figure 6
*C. sinensis*	[1] p. 433, plate 121, figure 7
*C. subhimalaspera*	[9] p. 81, figs. 3, 4
*C. subleptoceros*	[1] p. 501, plate 155, figs. 1, 3, 6

**Table 2:** Eight taxa of *Cymbella* producing internal pore occlusions.

Taxon	Shape of Internal Occlusions	Reference
*C. arctica*	Rounded closing plates	[1] p. 399, plate 104, figure 8
*C. balkii*	Manhole, rounded closing plate	[16] p. 198, figs. 14, 15
*C. cognata*	Likely reniform occlusion, eroded	[5] p. 499, figs. 79, 80
*C. cymbiformis*	Rounded closing plate	[1] p. 307, plate 58, figure 7; p. 309, plate 59, figs. 7, 8; p. 311, plate 60, figure 2; p. 319, plate 64, 4–6; p. 333, plate 71, figs. 7, 8
*C. nepalensis*	Rounded closing plate	[17] p. 330, figure 4e
*C. orientalis*	Rounded closing plate	[4] p. 102, figs. 20, 21
*C. orientalis* var. *delicatula*	Rounded closing plate	[12] p. 460, figs. 16–19
*C. schimanskii*	Rounded closing plate	[1] p. 349, plate 79, figure 6

**Table 3:** Comparison of features between *Cymbella apiculatophora* sp. nov. and similar taxa.

Feature	*C. apiculatophora*	*C. neuquina*	*C. neuquina* var. *fastigata*	*C. orientalis*	*C. orientalis* var. *delicatula*
Valve outline	Moderately dorsiventral	Moderately dorsiventral	Moderately dorsiventral	Weakly dorsiventral	Weakly dorsiventral
Apices	Apiculate	Bluntly cuneate	Cuneately rounded	Narrowly rounded	Rounded cuneate, subrostrate
Valve dimensions (μm)	Length 38–62, width 8–12	Length 58–127, width (17)19–24	Length 50–100, width 14.5–18.5	Length 17.5–46, width 5.5–8.5	Length 17.6–26.8, width 6–7.4
Central area	Trapezoid, more evident on dorsal side	Rounded, more evident on ventral side	Rounded, more evident on ventral side	Unilateral, transversely rectangular	Unilateral, transversely rectangular
Striae in 10 µm	11–12 (ventral)	6–8 (dorsal and ventral)	7–8 (dorsal and ventral)	11–12 (dorsal), 10–12 (ventral)	9–12 (dorsal), 10–13 (ventral)
Areolae in 10 µm	18–24	14–16	14–18	Ca. 25	28–32
Apical pore field	Composed of two unequal areas	One undivided area	No data	Composed of two unequal areas	Composed of two unequal areas
No. of stigmata	4–6	1–4	1–4	No stigma	No stigma
Reference	This paper	[19]	[19]	[4]	[12]

***Cymbella* cf. *excisiformis*** Krammer (Figure 5, Figure 6, Figure 7 and Figure 8).

**Table 4:** Comparison of features between *Cymbella hunanensis* sp. nov. and similar taxa.

Feature	*C. hunanensis*	*C. stigmaphora*	*C. subleptoceros*
Valve outline	Slightly dorsiventral, almost rhombic–lanceolate	Slightly dorsiventral, rhombic–lanceolate	Slightly dorsiventral, lanceolate
Apices	Cuneate, obtuse, not protracted	Acutely rounded	Narrowly rounded to acuminate– rounded
Valve dimensions (μm)	Length 32–58, width 8.5–12.5,	Length 27–57, width 10.7–14	Length 17–45, width 7.5–10
Central area	Elliptical	Absent	Absent
Striae in 10 µm	10–12 (dorsal), 11–13 (ventral)	9–13 (dorsal and ventral)	9–11 (dorsal and ventral)
Areolae in 10 µm	20–25	20–24	22–25
Stigmata	Absent	Absent	Absent
Intermissio	Clearly visible, ca. 1.5 μm long	No data	Clearly visible
Reference	This paper	[1]	[1]

***Cymbella hustedtii*** Krasske (Figure 12, Figure 13, Figure 14 and Figure 15).

**Table 5:** Comparison of features between *Cymbella juglandis* and similar taxa.

Feature	*C. juglandis*	*C. shii*	*C. subleptoceros*
Valve outline	Slightly dorsiventral, almost lanceolate	Dorsiventral, rhomboid–lanceolate	Slightly dorsiventral, lanceolate
Apices	Acuminate	More or less obtusely rounded	Narrowly to acuminate–rounded
Valve dimensions (μm)	Length 28–75, width 8–12,	Length 46–88, width 15–18	Length 17–45, width 7.5–10
Striae in 10 µm	10–12 (dorsal), 10–13 (ventral)	8–9 (dorsal and ventral)	9–11 (dorsal and ventral)
Areolae in 10 µm	22–27	15–19	22–25
Stigmata	Absent	Absent	Absent
Intermissio	Obscured	No data	Clearly visible plate 155, Figure 2
Reference	This paper	[23]	[1]

***Cymbella menyuanensis*** Bing Liu & Rioual sp. nov. (Figure 20, Figure 21, Figure 22 and Figure 23).

**Table 6:** Comparison of features between *Cymbella menyuanensis* sp. nov. and similar taxa.

Feature	*C. menyuanensis*	*C. neocistula*	*C. nepalensis*	*C. proxima*
Valve outline	Strongly dorsiventral	Strongly dorsiventral	Strongly dorsiventral	Strongly dorsiventral
Apices	Rostrate to subcapitate slightly bent towards dorsal side	Not protracted rounded	Broadly rounded	Not protracted rounded
Valve dimensions (μm)	Length 46–91, width 12.5–20.5	Length 34–110, width 12–19	Length 37–118, width 15–27	Length 38–120, width 18–24
Striae in 10 µm	8–11 (dorsal and ventral)	7–9 (dorsal and ventral)	8–10 (dorsal and ventral)	7–10 (dorsal and ventral)
Areolae in 10 µm	22–26	17–20	18–20	14–18
No. of stigmata	3–6	3–5	4–6	2–5
Reference	This paper	[1]	[9,17]	[1]

**The perizonia of two *Cymbella* species.**

**Table 7:** Shapes and sizes of median-size areolae in five *Cymbella* species.

Taxon	Shape and Size of External Areola Openings	Shape and Size of Internal Areola Openings	Shape and Size of the Occlusions of Internal Areola Openings
*C. apiculatophora*	Rounded (diameter ca. 250 nm) or dumbbell-shaped (ca. 200 nm long and 120 nm wide)	Oblong, ca. 240 nm long and 200 nm wide	Rounded solid plate without strut, diameter ca. 200–240
*C.* cf. *excisiformis*	Lineolate, ca	Rounded,	No occlusion
*C. hunanensis*	Reniform, ca. 420 nm long and 100 nm wide	Reniform, ca. 470 nm long and 240 nm wide	Reniform solid plate with one strut
*C. hustedtii*	Reniform, ca. 410 nm long and 100 nm wide	Reniform, ca. 380 nm long and 250 nm wide	Reniform solid plate with one strut
*C. menyuanensis*	Lineolate, ca. 580 nm long and 200 nm wide	Mushroom-shaped, ca. 160 nm long	Mushroom-shaped solid plate with one strut
*C. juglandis*	Slit-like or oblong, ca. 340 nm long and 100 nm wide	Elliptical, ca. 280 nm long and 250 nm wide	Shaped like walnut kernels (vola)

## Data Availability

The original contributions presented in the study are included in the article.

## References

[1] Krammer K., Lange-Bertalot H. (2002). Cymbella. Diatoms of Europe, Diatoms of the European Inland Waters and Comparable Habitats.

[2] Guiry M.D., Guiry G.M. (2024). AlgaeBase.

[3] Liu B., Zhou Y.Y., Blanco S., Williams D.M. (2022). Three new species of *Delicatophycus* M.J. Wynne (Bacillariophyta) from China, all possessing apical pore fields. Fottea.

[4] Lee J.H., Gotoh T., Chung J. (1993). *Cymbella orientalis* sp. nov., a freshwater diatom from the Far East. Diatom Res..

[5] Rodionova Y.V., Pomazkina G.V., Makarevich O.Y. (2013). *Encyonema mirabilis*, *Cymbella olgae* and *C. cognata*: New diatom species from Lake Baikal. Diatom Res..

[6] Glushchenko A.M., Maltsev Y.I., Kociolek J.P., Kuznetsova I.V., Kulikovskiy M.S. (2022). Molecular and morphological investigations of two giant diatom *Cymbella* species from the Transbaikal Area (Russia, Siberia) with comments on their distributions. Plants.

[7] Mirzahasanlou J.P., Qarebesloum T., Farasati M., Bahalkeh A. (2024). *Cymbella golestanica* sp. nov. a new diatom species from Agh Su Waterfall Golestan National Park, Northeastern Iran. Phytotaxa.

[8] Edlund M.B., Kheiri S., Mirzahasanlou J.P. (2024). Nomenclature and biogeography of the genus *Qinia* (Cymbellaceae, Bacillariophyceae). Not. Algarum.

[9] Jüttner I., Gurung S., Sharma C., Sharma S., De Haan M., Van de Vijver B. (2010). Morphology of new taxa in the *Cymbella aspera* and *Cymbella neocistula* groups, *Cymbella yakii* sp. nov. and *Cymbella* cf. *hanzschiana* from Everest National Park, Nepal. Pol. Bot. J..

[10] Le Cohu R., Lange-Bertalot H., Van de Viver B., Tudesque T. (2020). Analysis and critical evaluation of structural features in four Cymbellaceae taxa from New Caledonia. Fottea.

[11] Garcia M., Dutra D.B. (2016). *Cymbella loescherae* sp. nov. (Bacillariophyceae) from first-order streams of southern Brazil. Pol. Bot. J..

[12] Stancheva R., Ivanov P. (2011). *Cymbella orientalis* var. *delicatula* var. nov. (Bacillariophyta), a new epilithic stream diatom from Bulgaria. Nova Hedwig..

[13] Krammer K., Helmcke J.-G., Krammer K. (1982). Valve Morphology in the Genus *Cymbella* C.A. Agardh. Micromorphology of Diatom Valves.

[14] Bahls L. (2019). *Cymbella fontinalis* sp. nov. (Bacillariophyta, Cymbellaceae) from springs in the Rocky Mountains of North America. Nova Hedwig..

[15] Liu B., Williams D.M., Li Y., Tang Z.S. (2020). Two new species of *Cymbella* (Bacillariophyceae) from China, with comments on their valve dimensions. Diatom Res..

[16] Solak C.N., Balkis-Ozdelice N., Yilmaz E., Durmus T., Blanco S. (2021). Description of two new *Cymbella* (Bacillariophyta) species from Sakarbaşı spring, Turkey. Phytotaxa.

[17] Vishnyakov V.S., Kulikovskiy M.S., Dorofeyuk N.I., Genkal S.I. (2015). Morphology and Distribution of *Cymbella neocistula* Krammer and *Cymbella nepalensis* (Jüttner & Van de Vijver) Vishnyakov stat. nov. (Bacillariophyceae) in Water Ecosystems of South Siberia and Mongolia. Inland Water Biol..

[18] Moser G., Steindorf A., Lange-Bertalot H. (1995). Neukaledonien Diatomeenflora einer Tropeninsel. Revision der Collection Maillard und Untersuchungen neuen Materials. Bibl. Diatomol..

[19] Maidana N.I., Villanueva V.D., Krammer K. (2002). Taxonomy and valve structure of *Cymbella neuquina* Frenguelli (Bacillariophyceae), including a new combination, *C. neuquina* var. *fastigata* (Krasske) nov. comb. Nova Hedwig..

[20] Cox E.J. (2012). Ontogeny, homology, and terminology—Wall morphogenesis as an aid to character recognition and character state definition for pennate diatom systematics. J. Phycol..

[21] Novelo E., Tavera R., Ibarra C. (2007). Bacillariophyceae from Karstic Wetlands in Mexico. Bibl. Diatomol..

[22] Liu Y., Kociolek J.P., Kulikovskiy M., Glushchenko A., Yu P., Wang Q., Lu X., Fan Y. (2023). *Qinia* gen. nov. (Bacillariophyceae: Cymbellales) from Yunnan Province, China. J. Oceanol. Limnol..

[23] Gong Z.J., Li Y.L., Metzeltin D., Lange-Bertalot H. (2013). New species of *Cymbella* and *Placoneis* (Bacillariophyta) from late Pleistocene fossil, China. Phytotaxa.

[24] Kaczmarska I., Poulíčková A., Sato S., Edlund M.B., Idei M., Watanabe T., Mann D.G. (2013). Proposals for a terminology for diatom sexual reproduction, auxospores and resting stages. Diatom Res..

[25] Liu B., Williams D.M. (2020). From chaos to order: The life history of *Hannaea inaequidentata* (Lagerstedt) Genkal and Kharitonov (Bacillariophyta), from initial cells to vegetative cells. PhytoKeys.

[26] Bahls L., Luna T. (2018). *Cymbopleura laszlorum* spec. nov. (Cymbellaceae, Bacillariophyceae), a glacial relic from a calcium-rich floodplain fen in southwestern Montana, USA. Phytotaxa.

[27] Glushchenko A., Gusev E., Maltsev Y., Kociolek J.P., Kuznetsova I., Kulikovskiy M. (2021). *Cymbopleura natellia*—A new species from Transbaikal area (Russia, Siberia) described on the basis of molecular and morphological investigation. PhytoKeys.

[28] Kulikovskiy M., Lange–Bertalot H., Witkowski A., Dorofeyuk N. (2009). Morphology and taxonomy of selected cymbelloid diatoms from a Mongolian *Sphagnum* ecosystem with a description of three species new to science. Fottea.

[29] Harper M.A., Van De Vijver B., Kaulfuss U., Lee D.E. (2019). Resolving the confusion between two fossil freshwater diatoms from Otago, New Zealand: *Encyonema jordanii* and *Encyonema jordaniforme* (Cymbellaceae, Bacillariophyta). Phytotaxa.

[30] Kulikovskiy M., Kociolek J.P., Liu Y., Kuznetsova I., Glushchenko A. (2022). *Vladinikolaevia*, gen. nov.—A new enigmatic freshwater diatom genus (Cymbellaceae; Bacillariophyceae) from Mongolia. Fottea.

[31] Kapustin D.A., Kulikovskiy M., Kociolek J.P. (2017). *Celebesia* gen. nov., a new cymbelloid diatom genus from the ancient Lake Matano (Sulawesi Island, Indonesia). Nova Hedwigia, Beiheft.

[32] Liu B., Williams D.M., Liu Q.Y. (2018). A new species of *Cymbella* (Cymbellaceae, Bacillariophyceae) from China, possessing valves with both uniseriate and biseriate striae. Phytotaxa.

[33] Liu B., Blanco S., Lan Q.Y. (2018). Ultrastructure of *Delicata sinensis* Krammer et Metzeltin and *D. williamsii* sp. nov. (Bacillariophyta) from China. Fottea.

[34] Glushchenko A., Kuznetsova I., Kociolek J.P., Kulikovskiy M. (2019). *Karthickia verestigmata* gen. et sp. nov.—An interesting diatom with frustular morphology similar to several different cymbelloid diatom genera. Phycologia.

[35] Thirouin K.R. (2021). Systematics of the Freshwater Cymbelloid Diatoms (Bacillariophyta): History, Taxonomy and Phylogenetic Relationships. Master’s Thesis.

[36] Kermarrec L., Ector L., Bouchez A., Rimet F., Hoffmann L. (2011). A preliminary phylogenetic analysis of the Cymbellales based on 18S rDNA gene sequencing. Diatom Res..

[37] Nakov T., Ruck E.C., Galachyants Y., Spaulding S.A., Theriot E.C. (2014). Molecular phylogeny of the Cymbellales (Bacillariophyceae, Heterokontophyta) with a comparison of models for accommodating rate variation across sites. Phycologia.

[38] Kezlya E., Glushchenko A., Maltsev Y., Gusev E., Genkal S., Kuznetsov A., Kociolek J.P., Kulikovskiy M. (2020). *Placoneis cattiensis* sp. nov.—A new diatom (Bacillariophyceae: Cymbellales) soil species from Cát Tiên National Park (Vietnam). Phytotaxa.

[39] Karthick B., Hamilton P.B., Kociolek J.P. (2013). An Illustrated Guide to Common Diatoms of Peninsular India.

[40] Ross R., Sims P.A. (1972). The fine structure of the frustule in centric diatoms: A suggested Terminology. Br. Phycol. J..

[41] Ross R., Cox E.J., Karayeva N.I., Mann D.G., Paddock T.B.B., Simonsen R., Sims P.A. (1979). An amended terminology for the siliceous components of the diatom cell. Nova Hedwig. Beih..

[42] Mann D.G., Ross R. (1981). Sieves and flaps: Siliceous minutiae in the pores of raphid diatoms. Proceedings of the 6th Diatom Symposium.

[43] Cox E.J. (2004). Pore occlusions in raphid diatoms—A reassessment of their structure and terminology with particular reference to members of the Cymbellales. Diatom.

[44] Williams D.M. (1986). Comparative morphology of some species of *Synedra* Ehrenb. with a new definition of the genus. Diatom Res..

[45] Liu B. (2023). The diatom genus *Ulnaria* (Bacillariophyta) in China. PhytoKeys.

